# Mutational inactivation of Apc in the intestinal epithelia compromises cellular organisation

**DOI:** 10.1242/jcs.250019

**Published:** 2021-01-27

**Authors:** Helena Rannikmae, Samantha Peel, Simon Barry, Takao Senda, Marc de la Roche

**Affiliations:** 1 Department of Biochemistry, University of Cambridge, Cambridge CB2 1GA, UK; 2 Discovery Science, BioPharmaceuticals R&D, AstraZeneca, Cambridge CB4 0WG, UK; 3 Bioscience, Oncology R&D, AstraZeneca, Cambridge CB2 0RE, UK; 4 Department of Anatomy, Graduate School of Medicine, Gifu University, Gifu 501-1194, Japan

**Keywords:** Intestinal epithelia, Organoids, Microtubule cytoskeleton, Adenomatous polyposis coli, APC, Wnt pathway

## Abstract

The adenomatous polyposis coli (Apc) protein regulates diverse effector pathways essential for tissue homeostasis. Truncating oncogenic mutations in Apc removing its Wnt pathway and microtubule regulatory domains drives intestinal epithelia tumorigenesis. Exuberant cell proliferation is one well-established consequence of oncogenic Wnt pathway activity; however, the contribution of other deregulated molecular circuits to tumorigenesis has not been fully examined. Using *in vivo* and organoid models of intestinal epithelial tumorigenesis we found that Wnt pathway activity controls intestinal epithelial villi and crypt structure, morphological features lost upon Apc inactivation. Although the Wnt pathway target gene *c-Myc* (also known as *Myc*) has critical roles in regulating cell proliferation and tumorigenesis, Apc specification of intestinal epithelial morphology is independent of the Wnt-responsive *Myc-335* (also known as *Rr21*) regulatory element. We further demonstrate that Apc inactivation disrupts the microtubule cytoskeleton and consequently localisation of organelles without affecting the distribution of the actin cytoskeleton and associated components. Our data indicates the direct control over microtubule dynamics by Apc through an independent molecular circuit. Our study stratifies three independent Apc effector pathways in the intestinal epithelial controlling: (1) proliferation, (2) microtubule dynamics and (3) epithelial morphology.

This article has an associated First Person interview with the first author of the paper.

## Abstract (precis)

**Highlighted Article:** APC control of intestinal epithelia structure and function is regulated through three independent effector pathways specifying cell proliferation, epithelial morphology and intracellular organisation.

## INTRODUCTION

**Fig. 1.**
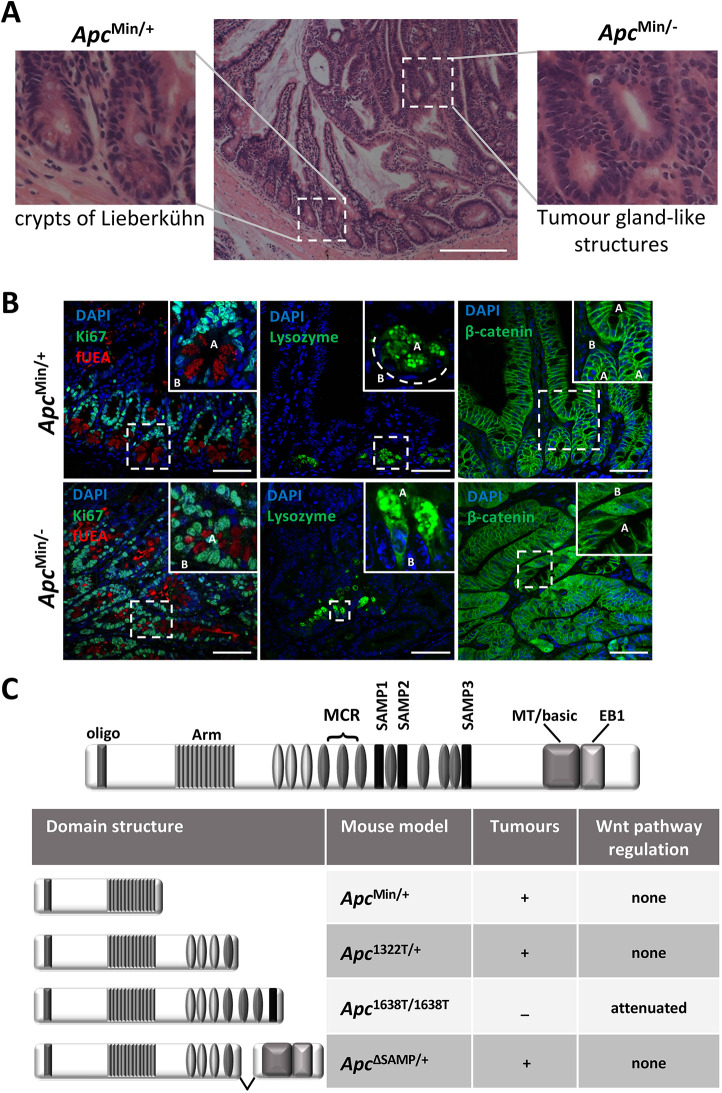
**Compromised morphology of the monolayer and cellular organisation in Apc-deficient intestinal epithelia.** (A) Haematoxylin and eosin stain of normal *Apc*^Min/+^ murine intestinal epithelia and adjacent *Apc*^Min/−^ tumour. The outsets show zoomed images of crypts from normal *Apc*^Min/+^ murine intestinal epithelia and gland-like structures from *Apc*^Min/−^ tumours. (B) Fluorescent confocal microscope imaging of sections of *Apc*^Min/+^ small intestinal epithelia (top panels) and *Apc*^Min/−^ tumours (bottom panels). The left panels show sections that are labelled with a fluorescent antibody against Ki67 (green). Intracellular vesicles are labelled with fluorescent fUEA (red). The middle panels show sections that are labelled with an antibody against lysozyme to mark Paneth cell vesicles. The right panels show sections that are labelled with an antibody against β-catenin. Nuclei in all sections are labelled with DAPI (blue). In the insets, ‘A’ marks the apical domain of cells and ‘B’ marks the basal domain. The curved dashed line delineates the monolayer of the base of the crypt. (C) Linear schematic of the Apc protein showing constituent protein-protein interaction domains. Arm, armadillo repeat domain; EB1, EB1-binding domain; MT/basic, the microtubule binding domain containing basic amino acids; Oligo, oligomerisation domain; SAMP1-3, Axin1-binding SAMP domains 1-3;. Ovals refer to 15- and 20-amino acid β-catenin binding domains (grey and dark grey, respectively). MCR marks the corresponding mutational cluster region in human APC. The table shows the mouse models of Apc deficiency used in this study, and domain structure of the expressed truncated forms of Apc alongside the genotype of the various models discussed in the main text. Additionally, the propensity for intestinal epithelial tumorigenesis in the mouse models, and the ability of the expressed truncated form of Apc to regulate Wnt pathway activity, is shown. Scale bars: 200 μm (A); 50 μm (B).

The intestinal tract (small intestine and colon) hosts a highly dynamic enterocyte monolayer that undergoes complete self-renewal every 3 to 5 days. The basic units of the intestinal epithelium are adjacent invaginations, termed crypts of Lieberkühn (Fig. 1A,B), each of which serves as a semi-autonomous cell production factory with a remarkably high proliferation rate – along the murine intestinal tract, crypts are composed of an average of 700 cells that produce up to 20 cells per hour in the small intestine or 7 cells per hour in the colon (de Rodriguez et al., 1978; Potten et al., 1982; Sunter et al., 1979). Throughout the enterocyte monolayer, each cell is spatially restricted, selective for homotypic and heterotypic cell-specific interactions, and is highly polarised with defined apical, lateral and basal faces, characteristics that are critical to epithelial barrier and transport functions. The hierarchal organisation of the enterocyte monolayer, from the stem cells at the base of crypts to differentiated cells types facing the gut lumen, is achieved through the highly regimented balance of rapid cellular proliferation, cellular organisation and morphology of the epithelial monolayer (Gehart and Clevers, 2019).

Malignant transformation as a result of mutational inactivation of the tumour suppressor gene adenomatous polyposis coli (*APC*) compromises tissue organisation of the intestinal epithelium (Dow et al., 2015; Kinzler and Vogelstein, 1996; Fearon and Volgestein, 1990). Somatic mutations in *APC* are widely regarded as the earliest genetic lesion in 80-90% of sporadic colon cancers (Groden et al., 1991). Perhaps surprisingly, mutational inactivation of APC reveals an oncogenic vulnerability largely restricted to the intestinal epithelium. Thus, individuals with familial adenomatous polyposis (FAP) that are heterozygous for a germline mutation inactivating one allele of *APC* (Su et al., 1992) exhibit spontaneous loss of heterozygosity that leads to hundreds of tumours, mostly restricted to the intestinal epithelium. The well-established murine model of FAP, *Apc*^Min/+^ (multiple intestinal neoplasia; Min), follows a similar pattern of tumour development – despite mono-allelic inactivation of *Apc* in every cell in the body, tumorigenesis is almost exclusive to the intestinal epithelium (Moser et al., 1990, 1995; Ren et al., 2019; Su et al., 1992).

Apc is a large multidomain protein that governs a plethora of effector pathways regulating cellular and tissue homeostasis (Nelson and Näthke, 2013). The molecular roles of Apc are generally ascribed to the regulation of Wnt pathway activity, a key determinant of stem cell multipotency and proliferation within the crypt. Pathway activity is sustained within the stem cell niche by redundant sources of Wnt ligands derived from adjacent Paneth cells and the underlying mesenchyme (Aoki et al., 2016; Farin et al., 2012; Gregorieff et al., 2005; Stzepourginski et al., 2017; Valenta et al., 2016; Zou et al., 2018), and potentiated by cellular engagement of LGR family receptors by R-spondins derived from specific mesenchymal cells (Yan et al., 2017).

Oncogenic APC inactivation in colorectal cancer follows a unique pattern of somatic changes – at least one APC allele harbours mutations that are largely confined to a short segment within exon 15 of the gene referred to as the mutation cluster region (MCR; Fig. 1C), resulting in the expression of truncated APC. The other allele is most often silenced or incurs the same or a more severe truncating mutations (Crabtree et al., 2003; Lamlum et al., 1999; Rowan et al., 2000). Tumours arising from truncating mutations in exon 14 of the mouse *Apc* gene found in the Min mouse line (Fig. 1C), equivalent to human exon 15, display many features common with human colorectal cancer tumours.

The truncated Apc protein lacks regulatory protein-protein interaction domains for the Wnt pathway regulators β-catenin (also known as Ctnnb1) and Axin1 (Fig. 1C), explaining oncogenic Wnt pathway activation upon loss of heterozygosity in murine models. Extensive investigation of oncogenic Wnt pathway activity in cells lacking Apc points to a key role in the regulation of intestinal epithelial cell proliferation through the Wnt pathway target gene c-*Myc* (Dave et al., 2017; He et al., 1998; Oskarsson and Trumpp, 2005; Sansom et al., 2007; Sur et al., 2012)*.*

Truncated Apc protein also lacks the C-terminal microtubule end binding protein 1 (EB1, also known as Mapre1) binding domain and a basic domain thought to bind directly to microtubules (Fig. 1C; Deka et al., 1998). However, the molecular consequence of C-terminal Apc truncations and removal of the microtubule and EB1 binding domains is controversial. Apc-mediated stabilisation of microtubules via its C-terminal domains supports the establishment of parallel arrays of microtubules in a polarised cell (Mogensen et al., 2002; Zumbrunn et al., 2001), and Apc is known to regulate cytoskeletal rearrangements that accompany cell motility, cell division and tissue organisation through control of microtubule dynamics (Moseley et al., 2007; Munemitsu et al., 1994; Näthke et al., 1996; Smith et al., 1994). Consistent with these observations is that Apc localizes to the plus-end tips of microtubules, centrosomes and microtubule spindles, and at the interface of microtubules and kinetichores during mitosis (reviewed by Näthke, 2004a). It is not clear whether the truncating mutations in Apc decrease its binding to microtubules or its capacity to stabilize the microtubule plus ends (Kroboth et al., 2007; Munemitsu et al., 1994; Smith et al., 1994; Zumbrunn et al., 2001). Furthermore, loss of Apc C-terminal microtubule and EB1 interaction domains in mouse embryonic fibroblasts and differentiated embryonic stem cells does not affect the distribution of β-tubulin (also known as Tubb1), EB1 and Apc (Lewis et al., 2012; Smits et al., 1999).

*In vivo* mouse models have investigated whether loss of the C-terminal microtubule and EB1 binding domains of Apc are sufficient to drive intestinal epithelial tumorigenesis. *Apc*^1638T/1638T^ mice express a version of Apc lacking the C-terminal domains but retaining the ability to regulate Wnt pathway activity, and do not present with intestinal epithelial tumours (Smits et al., 1999; Fig. 1C). Conversely, *Apc*^ΔSAMP/+^ mice, expressing a version of Apc unable to regulate Wnt pathway activity but retaining the microtubule and EB1 binding domains, develop tumours with the same frequency and kinetics as the corresponding *Apc*^1322/+^ mice that express Apc lacking these domains (Lewis et al., 2012; Fig. 1C). Thus, the ability of Apc to interact with microtubules and EB1 does not, on its own, drive intestinal epithelial tumorigenesis. Nonetheless, the potential contribution of the loss of the Apc microtubule and EB1 binding domains to intestinal tumorigenesis has not been determined.

Herein, we stratify molecular pathways regulated by Apc in the murine intestinal epithelia by defining the molecular and phenotypic consequences in the small intestinal epithelia and corresponding organoids caused by Apc inactivation. In addition to the deregulation of cell proliferation, we find that Apc inactivation disrupts the morphology of the intestinal epithelial monolayer and compromises the functional integrity of the microtubule cytoskeleton in component enterocytes. These emergent malignant properties are the direct consequence of Apc inactivation and are controlled by different and independent molecular systems. Therefore, (1) enterocyte proliferation, (2) microtubule dynamics and (3) epithelial morphology are regulated by three separate effector pathways that, under the control of Apc, buttress normal intestinal epithelial homeostasis against malignant transformation.

## RESULTS

### Compromised intracellular organisation and tissue morphology in *Apc*^Min/−^ tumours

Over the course of 110 days, *Apc*^Min/+^ mice develop 30-40 adenomas in the small intestine, the result of loss of heterozygosity of the wild-type *Apc* allele (Moser et al., 1995; Su et al., 1992). Such *Apc*^Min/−^ tumours are composed of gland-like structures that maintain an epithelial monolayer yet lack the morphological hallmarks of crypt and villus compartments, and the hierarchal cellular organisation of the wild-type epithelia (Fig. 1A). For instance, Ki67^+^ proliferative stem cells and the transit amplifying cellular compartment, normally disposed basally within crypts, are instead interspersed throughout the monolayer of the tumour gland-like structures (Fig. 1B). We used fluorescently-labelled *Ulex europaeus* agglutinin (fUEA) to visualise secretory vesicles that, in wild-type tissue, are found apically within the mechanically rigid keystone-shaped Paneth cells in the crypts or columnar-shaped goblet cells in the villi (Langlands et al., 2016; Fig. 1B). However, UEA^+^ cells in *Apc*^Min/−^ tumours were interspersed throughout the glandular monolayer, were of variable shapes and failed to maintain their characteristic clustered apical localisation (Fig. 1B). We observed the identical phenotype using an antibody raised against lysozyme – Paneth cells no longer maintained their shape, size and clustered localisation to the crypt base, and intracellular secretory vesicles were no longer apically restricted (Fig. 1B). In contrast, β-catenin maintained its characteristic localisation pattern at the cell periphery juxtaposed to cell-cell contacts in *Apc*^Min/−^ tumour cells (Fig. 1B). In the larger proportion of *Apc*^Min/−^ tumour cells, we observed high expression levels of β-catenin and localisation to the nucleus, an established consequence of Apc deficiency (Polakis, 2012). In general, tumour cells failed to maintain the consistent columnar cell shape and size of wild-type epithelial cells, yet they aligned as a monolayer to form the gland-like structures (Fig. 1A,B). We also note that, as opposed to wild-type enterocytes, *Apc*^Min/−^ tumour cells contained nuclei of variable shapes and sizes that failed to align along the plane of the monolayer. We conclude that, in addition to driving deregulated epithelial cell proliferation and the disruption of tissue morphology, Apc inactivation compromises some aspects of intracellular organisation.

### Defective regulation of microtubule function in *Apc*^Min/−^ tumours

**Fig. 2.**
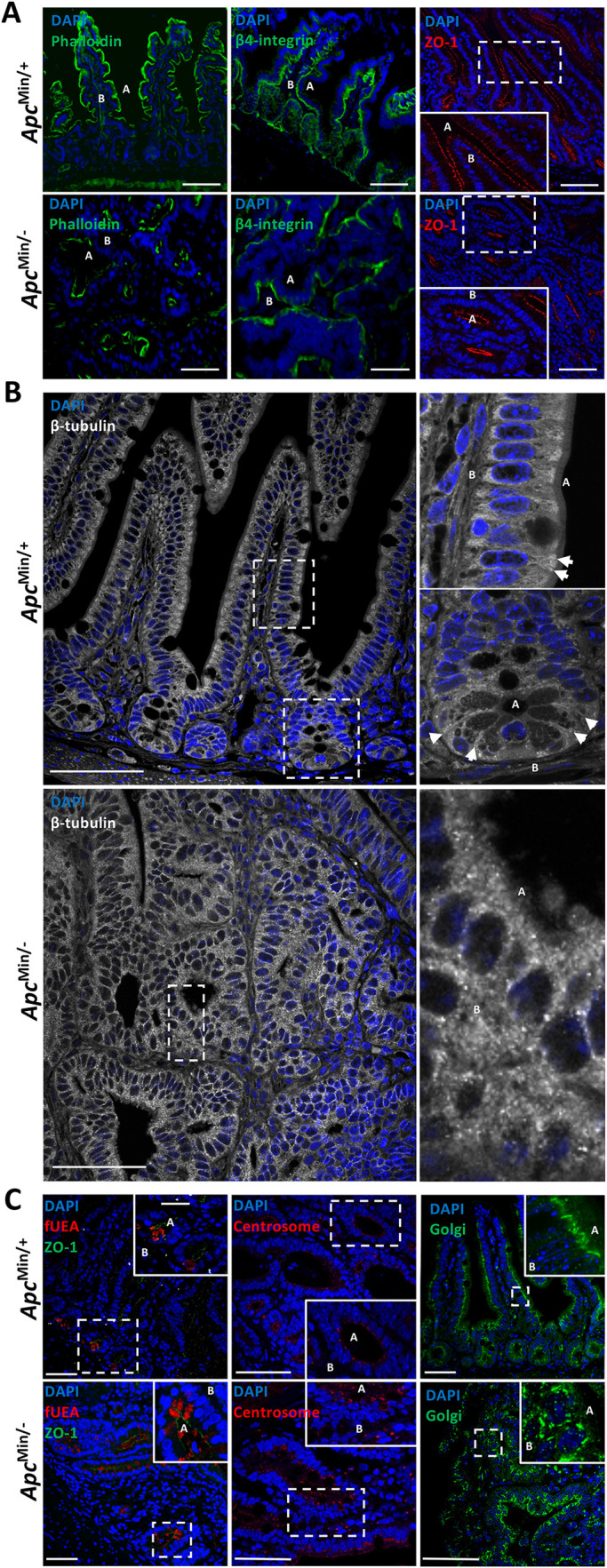
**Apc inactivation does not affect the localisation of the actin cytoskeleton yet compromises microtubule dynamics.** (A) Fluorescence confocal microscopy of small intestinal epithelial sections from *Apc*^Min/+^ mouse (top panels) and *Apc*^Min/−^ tumours (bottom panels). Sections were labelled with fluorescent phalloidin (green; left panel), an antibody against β4-integrin (green; middle panel) and an antibody against ZO-1 (red; right panel). (B) Confocal images of sections from *Apc*^Min/+^ mouse (top panels) and *Apc*^Min/−^ tumours (bottom panels) labelled with an antibody against β-tubulin. The top right panels are zoomed images of crypt and villi regions and the bottom right panel is a zoomed image of a region of the tumour. Arrowheads indicate the position of individual microtubules aligning along the apical-basal axis of epithelial cells. (C) Sections of small intestinal epithelia from *Apc*^Min/+^ mouse (top panels) and *Apc*^Min/−^ tumours (bottom panels). The left panels show sections labelled with fluorescent UEA (red) and an antibody against ZO-1 (green). The middle panels show sections labelled with an antibody against pericentrin (red) to visualise the centrosome. The right panels show sections labelled with an antibody against the Golgi resident protein ZFPL1 (green). All sections were co-labelled with DAPI (blue). In all panels, ‘A’ marks the apical domain of cells and ‘B’, the basal domain. Scale bars: 50 μm (A,C); 100 μm (B).

The cytoskeleton provides the physical framework for intracellular organisation and cell polarity defined by localised dynamic polymerisation/depolymerisation of actin and tubulin monomers (Li and Gundersen, 2008; Rodriguez-Boulan and Macara, 2014). Apc harbours an array of protein-protein interaction domains with established roles in regulating F-actin and microtubule dynamics within intestinal epithelial cells (Fig. 1C) (Kawasaki et al., 2000; Munemitsu et al., 1994; Näthke, 2004b; Rosin-Arbesfeld et al., 2001; Tirnauer, 2004; Zumbrunn et al., 2001). We examined the localisation of the cytoskeleton in intestinal epithelial and *Apc*^Min/−^ tumour cells using a series of fluorescent probes for F-actin, microtubules and known protein interactors. Consistent with a previous study (Fatehullah et al., 2013), all *Apc*^Min/−^ tumour cells maintained the correct disposition and configuration of actin cytoskeletal components – F-actin was concentrated along the apical face of the epithelial cells (Pelaseyed and Bretscher, 2018), β4-integrin (also known as Itgb4), which anchors enterocytes to the underlying lamina propria, was found at the cell base (Fatehullah et al., 2013), β-catenin localised adjacent to cell-cell contacts and the tight junction organiser ZO-1 (also known as Tjp1) was positioned apically at cellular junctions (Lee et al., 2018; Fig. 1B, Fig. 2A, Table 1).

**Table 1.**
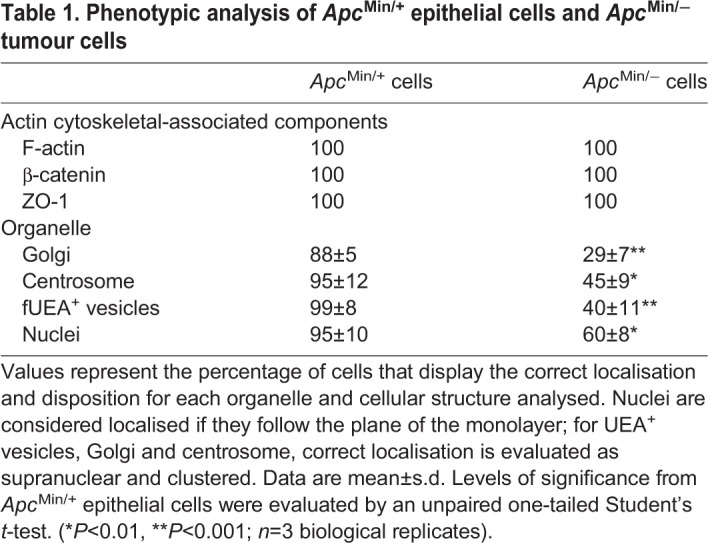
**Phenotypic analysis of *Apc*^Min/+^ epithelial cells and *Apc*^Min/−^ tumour cells**

In contrast, components of the microtubule cytoskeleton in *Apc*^Min/−^ tumour cells were disorganised; microtubules, normally orientated along the apical-basal axis were instead disjointed and diffuse, often appearing as punctate (Fig. 2B). We used an antibody raised against the acetylated form of α-tubulin and found that the signal was concentrated at the apical domain of cells, in line with previously published data (Quinones et al., 2011). However, in tumour cells, acetylated α-tubulin was instead delocalised and diffuse (Fig. S1A). As a quantitative measure of the functional microtubule cytoskeleton, we determined the localisation of intracellular organelles whose location and positioning are dependent on microtubules. Predictably, we found that the normally strict basal positioning of nuclei, apical positioning of intracellular vesicles and the supranuclear localisation of the Golgi resident protein ZFPL1 and the centrosome marker pericentrin in wild-type intestinal epithelia was lost in *Apc*^Min/−^ tumour cells. Instead we observed signals for the Golgi and centrosome split into multiple puncta and mislocalised (Fig. 2C, Table 1). To preclude mislocalisation of the Golgi as the consequence of cells undergoing cell division (Thyberg and Moskalewski, 1999), we co-stained intestinal epithelial sections with an antibody against the mitotic marker phospho-histone 3 (PH3) – tumour cells displayed delocalised Golgi in the absence of detectable levels of PH3 (Fig. S1B). We quantified localisation data for components associated with the microtubule and actin cytoskeleton (presented in Table 1). Our results indicate that normal localisation of the actin cytoskeleton is maintained in *Apc*^Min/−^ intestinal epithelial tumour cells, whereas the localisation and functional integrity of the microtubule cytoskeleton is compromised.

We reasoned that the C-terminal microtubule and EB1 binding domains of Apc may be critical for the regulation of the microtubule cytoskeleton. The *Apc*^1638T/1638T^ mouse strain is homozygous for a truncating mutation in Apc that deletes the C-terminal microtubule and EB1 binding domains (Fig. 1C). However, as opposed to Apc expressed in *Apc*^Min/+^ mice, the Apc^1638T^ protein retains the Axin1 interaction domain and therefore retains regulatory control over Wnt pathway activity; *Apc*^1638T/1638T^ mice do not develop intestinal epithelial tumours (Smits et al., 1999; Fig. 1C). As the small intestine epithelia of *Apc*^1638T/1638T^ mice exhibit normal localisation of intact Golgi and fUEA^+^ Paneth cell vesicles (Fig. S1C), we conclude that loss of the C-terminal microtubule and EB1 binding domains of Apc alone do not compromise regulation of the microtubule cytoskeleton or intestinal epithelial morphology.

### Organoids accurately recapitulate the molecular and phenotypic consequences of APC inactivation in the intestinal epithelium

We generated organoid lines from wild type, *Apc*^Min/+^ intestinal epithelia and *Apc*^Min/−^ tumour cells as an experimentally tractable model system for determining the molecular mechanisms linking Apc to microtubule integrity and epithelial morphology. Organoids derived from normal tissue form an epithelial monolayer, replete with crypts, that maintains the three-dimensional cellular organisation and hierarchy found *in vivo*. In contrast, tumouroids, organoids derived from *Apc*^Min/−^ tumour cells, form cystic structures lacking morphological features of the intestinal epithelial monolayer, such as crypts (Sato et al., 2011).

**Fig. 3.**
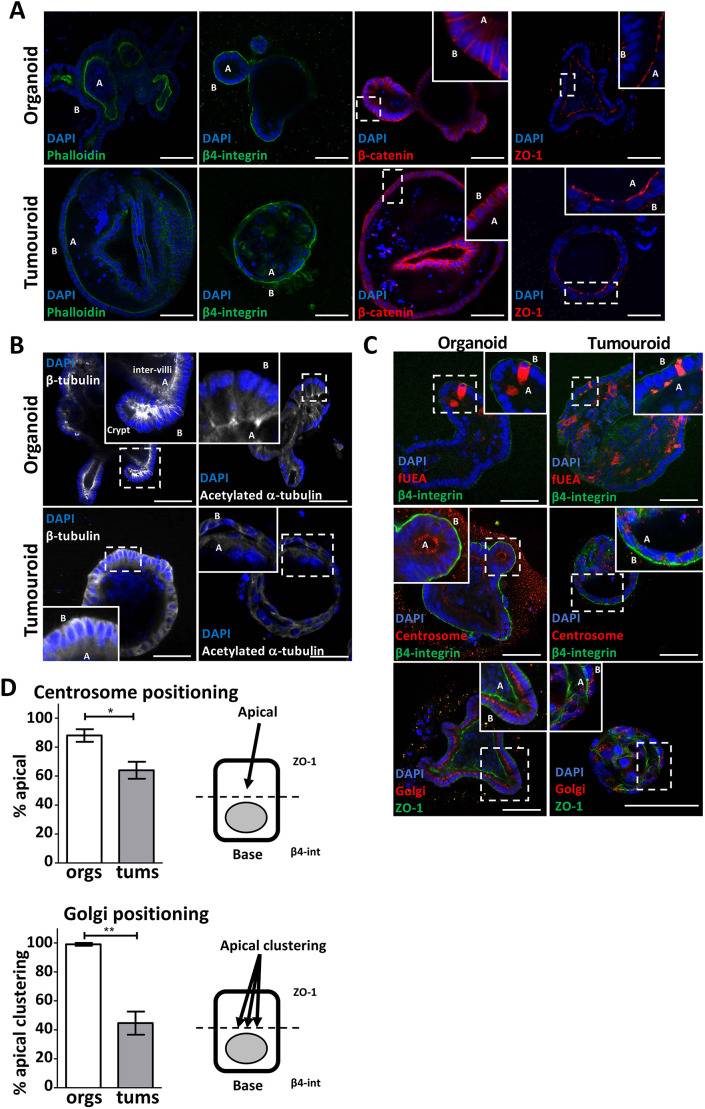
**Organoids recapitulate the consequences of Apc inactivation in the intestinal epithelia.** (A) Fluorescence confocal microscopy of small intestinal epithelial organoids (top panels) and *Apc*^Min/−^ tumouroids (bottom panels). All cells were labelled with DAPI (blue) and fluorescent phalloidin (green; left panel), or antibodies against β4-integrin (green; middle left panel), β-catenin (red; middle right panel) and ZO-1 (red; right panel). ‘A’ marks the apical domain of cells in the monolayers and ‘B’ marks the basal domain. (B) Organoids (top panels) and *Apc*^Min/−^ tumouroids (bottom panels) were labelled by immunofluorescence using antibodies against β-tubulin and acetylated-α-tubulin, as marked. All cells were labelled with DAPI (blue). ‘A’ marks the apical domain of cells in the monolayers and ‘B’ marks the basal domain. (C) Immunofluorescence of small intestinal epithelial organoids (left panels) and *Apc*^Min/−^ tumouroids (right panels). The top panels show labelling with fUEA (red) and an antibody against β4-integrin (green). Middle panels show labelling with antibodies against pericentrin (red) and an antibody against β4-integrin (green). Bottom panels show labelling with antibodies against ZFPL1 (red) and an antibody against ZO-1 (green). All cells were labelled with DAPI (blue). ‘A’ marks the apical domain of cells in the monolayers and ‘B’ marks the basal domain. (D) The cellular positioning of the centrosome and the Golgi was scored as apical or apical and clustered in organoids (orgs) or tumouroids (tums), according to the schematics in the right panels for >200 cells from three independent experiments. Data are mean±s.d. Statistical differences in centrosome and Golgi localisation in tumouroids relative to organoids were determined by an unpaired one-tailed Student's *t*-test (*P<0.01, **P<0.001). Scale bars, 50 μm.

Using a series of fluorescent probes, we found that F-actin and associated molecular components β4-integrin, β-catenin and ZO-1 maintained their intracellular localisation in both organoid and tumouroid cells (Fig. 3A). Note that in some cases, the middle portion of the spherical tumouroid collapses and is captured in some confocal sections. Consistent with our observations in intestinal epithelial tissue from *Apc*^Min/−^ tumours, the organisation and function of the microtubule cytoskeleton was compromised: β-tubulin was no longer polarised in microtubules along the apical-basal axis of cells but was instead dispersed throughout tumouroid cells and acetylated α-tubulin was delocalised (Fig. 3B). We also found that nuclei varied in shape and size and did not follow the plane of the tumouroid monolayer and centrosomes, and Golgi were split into multiple puncta distributed throughout the cell body (Fig. 3C). Quantification of the centrosome and Golgi localisation in tumouroids indicated that they were mispositioned in over 35% and 50% of cases, respectively (Fig. 3D). Consistent with our *in vivo* results, our organoid data confirm that Apc inactivation in the intestinal epithelial monolayer leads to deregulation of microtubule dynamics and loss of intracellular organisation with the absence of detectable effects on the actin cytoskeleton.

### Apc deficiency directly compromises intracellular organisation and tissue morphology

It is possible that intestinal epithelial tumours from 110-day-old *Apc*^Min/+^ mice, and organoids derived from them, have acquired additional somatic changes that contribute to phenotype. To determine the immediate and direct effects of Apc inactivation, we created a switchable organoid model of tumorigenesis that relies on the inducible expression of a previously validated shRNA targeting Apc (Dow et al., 2015) (Fig. S2A). Induction of shApc in organoids depleted Apc mRNA concurrent with the expression of mCherry, and leads to the intraconversion of organoids into a cystic tumouroid structure (Fig. S2B-D). Importantly, we observed a reversible increase in expression of the Wnt pathway target gene *c-Myc* (Fig. S2E).

**Fig. 4.**
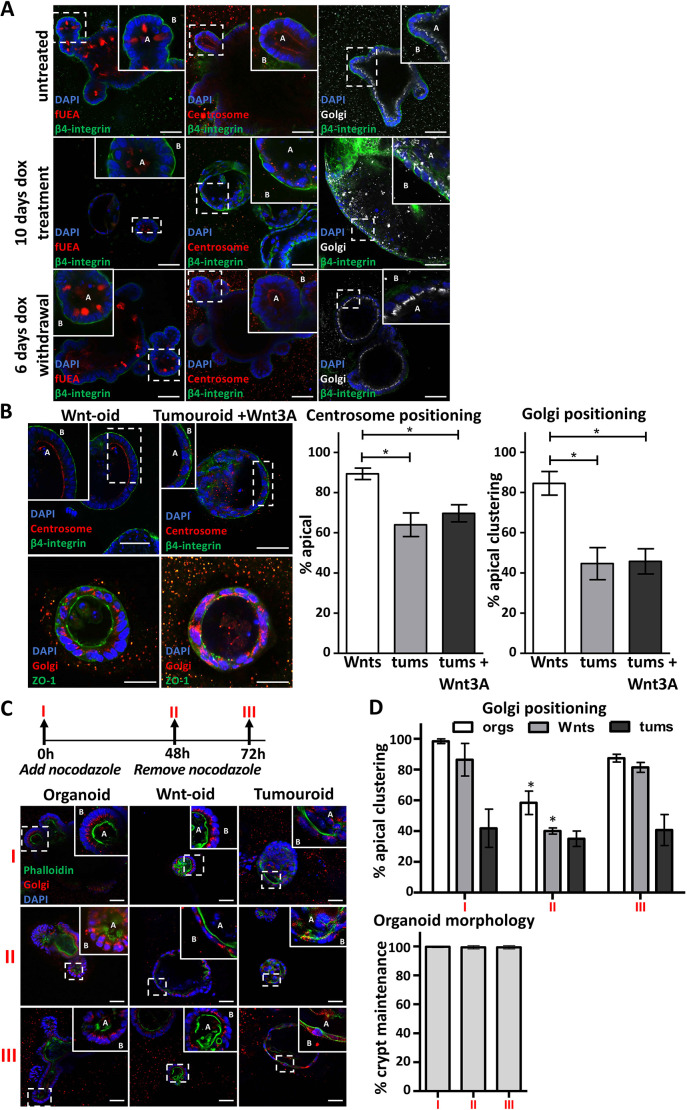
**Switchable *in vitro* model of tumorigenesis recapitulates the consequences of Apc inactivation in the intestinal epithelia.** (A) An organoid line bearing pB-shApc, the Tet-on inducible transgene system for the induction of shApc expression (Fig. S2), untreated (top panels), treated with doxycycline for 10 days (middle panels), or the former followed by doxycycline withdrawal for an additional 6 days (lower panels). The left panels show fluorescence confocal microscopy of organoids labelled with fUEA (red). The middle panels show organoids labelled with antibodies to pericentrin (centrosome, red). The right panels show organoids labelled with antibodies against ZFPL1 (Golgi, white). All specimens were co-labelled with an antibody against β4-integrin (green) and DAPI (blue). ‘A’ marks the apical domain of cells in the monolayers and ‘B’ marks the basal domain. (B) Fluorescence confocal microscopy of organoids (left panels) and tumouroids (right panels) treated with Wnt3A-conditioned medium for 72 h and labelled with antibodies as marked. The top panels show specimens co-labelled with antibodies against pericentrin (centrosome, red) and β4-integrin (green). The bottom panels show specimens co-labelled with antibodies against ZFPL1 (Golgi, red) and ZO-1 (green). All specimens were co-labelled with DAPI (blue). The right panels show graphs quantifying apical localisation of centrosome and apical clustering of Golgi for the Wnt-oids and tumouroids treated with Wnt3A. More than 200 cells from three independent fluorescent sections were analysed according to the schematic in Fig. 3D. (C) Top: schematic depicting the nocodazole treatment regime for organoid, Wnt-oid and tumouroid cultures. The panels below show fluorescence confocal microscopy of cultures treated as follows: control (I); treated with nocodazole (II); or nocodazole treatment followed by drug withdrawal for 72 h (III). All panels show labelling with fluorescent phalloidin (green) and DAPI (blue), and probed with an antibody against ZFPL1 (Golgi, red). ‘A’ marks the apical domain of cells in the monolayers and ‘B’ marks the basal domain. (D) Top: quantification of apical Golgi clustering (according to the schematic in Fig. 3D). Bottom: percentage of organoid morphology among the nocodazole treatment groups quantified as the percentage of organoids that maintained two or more crypts under the treatment conditions. Data are mean±s.d. and derived from analysis of 50 organoids from two independent experiments. Statistical differences among organoid, Wnt-oid and tumouroid phenotypes (B), and for Golgi localisation for treated samples (D, II and III) relative to control (D, I) were calculated using an unpaired two-tailed Student's *t*-test (*P<0.001). There were no significant differences in organoid morphology among the treatment groups in D. Scale bars: 50 μm.

Consistent with the appearance of *Apc*^Min/−^ tumours and tumouroids, Apc depletion in organoids resulted in the mislocalisation of UEA^+^ intracellular vesicles, as well as Golgi and centrosome fragmentation and mislocalisation (Fig. 4A). Importantly, all hallmarks of intracellular disorganisation and compromised tissue morphology were reversed upon Apc re-expression, leading to the appearance of ‘normal’ organoids (Fig. 4A). Our switchable *in vitro* tumorigenesis model confirms that compromised epithelial morphology and intracellular disorganisation are the direct consequence of Apc inactivation.

### Apc regulation of intestinal epithelial morphology and microtubule dynamics are discrete

Ubiquitous activation of Wnt pathway activity in organoid cells by treatment with Wnt3A-conditioned medium leads to the intraconversion of organoids into cystic tumouroid-like structures (Farin et al., 2012) that we refer to as Wnt-oids (Fig. 4B). Although the morphology of the Wnt-oid epithelial monolayer is compromised, they are distinct from tumouroids in that the Golgi and centrosome retain their normal apical position in component cells (Fig. 4B) – greater than 80% of Wnt-oid cells show apical localisation of the Golgi and centrosome as opposed to less than 65% in tumouroid cells (Fig. 4B). We conclude that Apc regulation of intestinal epithelial morphology through Wnt pathway regulation is not coupled to its function in regulating microtubule dynamics and intracellular organisation.

We carried out the complementary experiment, selectively deregulating microtubule dynamics in organoids and determining the consequence on epithelial morphology. We treated organoids with a low concentration (100 nM) of the microtubule depolymerising agent nocodazole (Vasquez et al., 1997) for 48 h, a time point sufficient for the conversion of organoids to Wnt-oids with Wnt3A treatment. Treated organoid and Wnt-oid cells displayed the characteristic mislocalisation of fragmented Golgi that was reversed after 24 h post-nocodazole withdrawal (Fig. 4C,D). Importantly, throughout the experiments, nocodazole-treated organoids maintained intestinal epithelial crypt structures (Fig. 4C,D), indicating that maintenance of intestinal organisation and microtubule dynamics are not dependent on one another. Combined with our Apc loss-of-function studies, these data suggest that Apc-dependent control of intracellular organisation and epithelial morphology rely on independent molecular circuits.

### Loss of a Wnt-responsive enhancer element upstream of *c-Myc* does not impact intestinal epithelial morphology

Previous studies have indicated that Apc inactivation in the intestinal epithelia compromising Wnt pathway-dependent regulation of *c-Myc* expression is the critical mediator of malignant transformation *in vivo* (Dave et al., 2017; Sansom et al., 2006; Sur et al., 2012). Although the removal of the Wnt pathway-responsive enhancer element upstream of *c-Myc*, which carries binding sites for the Wnt pathway effector transcription factor Tcf7l2 (*Myc-335^−/−^* mice; Fig. S3) (Sur et al., 2012), only modestly reduces *c-Myc* expression, it attenuates small intestinal epithelial tumorigenesis, when combined with the *Apc*^Min/+^ allele, by ∼70% multiplicity (Sur et al., 2012). We derived organoids from *Myc-335^−/−^* mice to test whether deregulated epithelial morphology, one phenotypic consequence of Wnt pathway activity imposed by Apc inactivation, was altered after deletion of the Wnt-responsive enhancer element upstream of *c-Myc*.

**Fig. 5.**
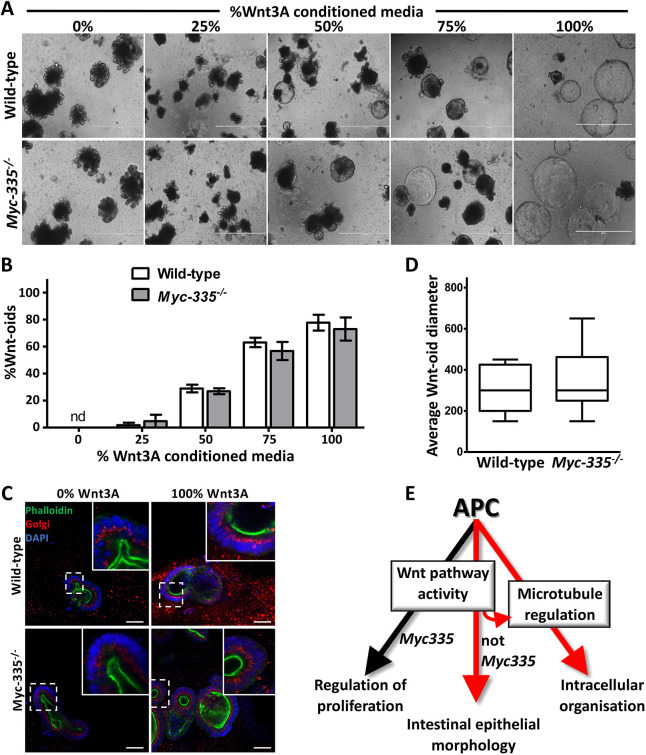
**The Wnt pathway target gene *c-Myc* does not specify tissue morphology and intracellular organisation upon Wnt pathway activation.** (A) Representative bright-field images of wild-type and *Myc-335*^−/−^ organoids grown in increasing concentrations of Wnt3A-conditioned medium for 7 days. (B) Quantification of Wnt-oid formation under conditions described above. Data displayed is derived from a minimum of 200 individual organoids from two independent experiments for each concentration of Wnt3A-conditioned medium. nd, no Wnt-oids detected. (C) Fluorescence confocal microscopy of wild-type and *Myc-335^−/−^* organoids grown in the absence or presence of the maximal dose of Wnt3A-conditioned medium for 7 days. All sections were labelled with DAPI (blue), fluorescent phalloidin (green) and an antibody against ZFPL1 (Golgi, red). (D) Growth rate measured as average Wnt-oid diameter (µM) of wild-type and *Myc-335*^−/−^ organoids after 7 days growth in 100% Wnt3A-conditioned medium. Data for the box plots was from more than 50 organoids from two independent experiments for each organoid type. Boxes show the interquartile range, with the median indicated. Whiskers show the range. (E) Model for Apc regulation of proliferation, tissue morphology and intracellular organisation in the small intestinal epithelia. Red arrows represent Apc effector pathways stratified in the current study. See text for details. Data are mean±s.d. No statistically significant differences were observed in the frequency of Wnt-oid morphology (B) or in growth rate between organoid genotypes (D), as evaluated using an unpaired two-tailed Student's *t*-test. Scale bars: 1000 μm (A); 50 μm (C).

Wnt3A-conditioned medium treatment of *Myc-335^−/−^* and wild-type organoids indicated identical kinetics and frequency of Wnt-oid formation (Fig. 5A,B) that retained the normal Golgi apical localisation (Fig. 5C). Furthermore, over the 7-day time course of Wnt3A treatment, we observed no differences in cell proliferation within wild-type and *Myc-335^−/−^* organoids measured by Wnt-oid diameter (Fig. 5D). Taken together, our data suggest that regulation of intracellular organisation and epithelial tissue morphology by Wnt pathway activity is independent of regulation of c-Myc expression via *Myc-335*.

## DISCUSSION

In this study, we unmasked individual molecular systems controlled by Apc in the intestinal epithelia through loss of function. Oncogenic Apc mutations are the principal driver of colon epithelial tumorigenesis and are sufficient for malignant transformation of the colon and small intestinal epithelia. We stratify three emergent phenotypes in the murine intestinal epithelia that are the direct consequence of oncogenic Apc mutations: deregulated proliferation, disrupted epithelial morphology and compromised microtubule dynamics leading to defective intracellular organisation.

In the intestinal epithelia, Apc activity restricts enterocyte proliferation through stringent control of the Wnt pathway-dependent transcriptional programme. In particular, regulated expression of the Wnt pathway target gene *c-Myc* constrains proliferation to discrete localised niches, providing a key molecular barrier to malignant transformation (Dave et al., 2017; Quyn et al., 2010; Sur et al., 2012); although oncogenic Apc mutations in the intestinal epithelia are sufficient to drive neoplastic growth, the absence of *c-Myc* expression attenuates all transforming properties of Apc inactivation *in vivo* (Sansom et al., 2006). Less well understood is how oncogenic Apc mutations deregulate epithelial morphology and intracellular organisation. We have established that organoids and their Apc-deficient counterparts, tumouroids, are a tractable model that effectively recapitulates the morphological and organisational hallmarks modelling the transition between intestinal epithelia and tumours.

Treatment of organoids with Wnt3A drives their intraconversion into cystic tumouroid-like structures, termed Wnt-oids, that, in contrast to tumouroids, maintain intracellular organisation of the component cells. Our interpretation is that Wnt3A treatment leads to selective inhibition of Wnt pathway regulation by Apc, compromising constraints on epithelial morphology but retaining the integrity of the microtubule cytoskeleton and intracellular organisation, supporting the notion that regulation of epithelial morphology and cytoskeletal integrity are uncoupled. Conversely, selective destabilisation of microtubules compromises intracellular organisation in component organoid cells, yet normal morphology of the epithelia monolayer is retained. Taken together, our data support a model whereby Apc controls enterocyte proliferation and epithelial morphology through Wnt pathway regulation, and regulates the microtubule cytoskeleton and intracellular organisation through other separate pathways (Fig. 5E).

How then does Apc regulation of Wnt pathway activity impact the morphology of the epithelial monolayer? Our data support direct control of epithelial morphology by Wnt pathway activity rather than an inability of organisational constraints to cope with exuberant proliferation. In the intestinal epithelia, neoplastic growth is the result of precocious Wnt pathway target gene expression driving deregulated expression of the Wnt pathway target gene *c-Myc*. Deregulated *c-Myc* expression is regarded as the major culprit in all transforming phenotypes attributed to Apc loss *in vivo* (Sansom et al., 2007). However, using the *Myc-335^−/−^* organoids, we find that although the modest deregulation of *c-Myc* expression observed *in vivo* (Sur et al., 2012) is sufficient to reduce transforming properties of Apc inactivation, it does not alter Wnt pathway regulation of intestinal epithelial morphology or cellular organisation in our organoid system. Moreover, within the time frame of our experiments, we did not observe any changes in the rate of proliferation accompanying the intraconversion of organoids to Wnt-oids. Together, our data suggest that Apc control of epithelial morphology is not wholly dependent on *Myc-335*-mediated Wnt pathway regulation of *c-Myc* expression, nor is it the result of increased proliferative pressure on organisational constraints on the epithelial monolayer.

It will be important to identify Wnt pathway targets that control intestinal epithelial morphology – we anticipate that targeted modulation of such genes may provide therapeutic value for preventing or even reversing the compromised epithelial morphology accompanying malignant transformation of the intestinal epithelia. The intraconversion between organoids and Wnt-oids is a ready-made assay system for rapidly testing sufficiency of Wnt pathway candidate target genes by their targeted loss of function; a list of such candidates has been previously identified by Sansom et al. (2007).

One striking observation was that Apc regulates the integrity of the microtubule cytoskeleton and consequently the intracellular location of organelles, such as the nucleus, Golgi, centrosome and intracellular vesicles. Although control of the microtubule cytoskeleton may be mediated directly by the Apc C-terminal microtubule and/or EB1 binding domains (Morrison et al., 1998; Munemitsu et al., 1994), it is also possible that Wnt pathway regulatory components downstream of Apc, or even Wnt pathway transcriptional targets, contribute to microtubule integrity. For example, truncated Apc in *Apc*^1638T/1638T^ mice retains the ability to regulate Wnt pathway activity and maintain the integrity of the microtubule cytoskeleton (Smits et al., 1999). Our interpretation is that regulation of the Wnt pathway suppresses defects in the microtubule cytoskeleton *in vivo*. It remains to be determined whether this is the case in the intestinal epithelial-autonomous milieu of *in vitro* organoid culture.

In colon cancer, oncogenic mutations that inactivate Apc are tenfold more prevalent than oncogenic mutations in other Wnt pathway regulatory components, suggesting that functions other than Wnt pathway deregulation contribute to disease aetiology. Although compromised microtubule integrity is a likely consequence of Apc truncations that delete C-terminal microtubule and EB1 binding domains, it is unlikely to impact tumorigenesis – the presence of C-terminal microtubule and EB1 binding domains in truncated versions of Apc has no impact on tumorigenesis (Lewis et al., 2012). However, one intriguing possibility is that compromised microtubule integrity in Apc mutant tumour cells contributes to chromosome instability (CIN). CIN is a feature of the evolution of aggressive colorectal adenocarcinoma right from the outset, being evident in the smallest adenomas, and multiple reports have directly linked oncogenic APC mutations in colorectal cancer with a predisposition to CIN (Dikovskaya et al., 2007; Fodde et al., 2001; Kaplan et al., 2001). Importantly, embryonic stem cells derived from *Apc*^1638T/1638T^ mice develop hallmarks of CIN (Fodde et al., 2001), and overexpression of truncated APC lacking the C-terminal domains in chromosomally stable colorectal cancer cells leads to mitotic defects, including errors in kinetochore attachment and alignment of chromosomes (Green and Kaplan, 2003; Tighe et al., 2004). However, the molecular relationship between Apc loss, microtubule deregulation and chromosome instability in the intestinal epithelia has yet to be established. The experimentally tractable organoid/tumouroid model system we have developed will be invaluable in determining the role of Apc in the loss of microtubule integrity and the impact of CIN in intestinal tumorigenesis.

Our results distinguish individual malignant properties of intracellular disorganisation, compromised tissue morphology and proliferation as direct but independent consequences of Apc inactivation; we posit that the combination of these emergent properties creates a ‘perfect storm’ for malignant transformation of the rapidly dividing intestinal epithelia, explaining why this tissue is particularly vulnerable to oncogenic Apc mutations.

## MATERIALS AND METHODS

### Reagents, antibodies and molecular probes

Doxycycline and nocodazole were sourced from Sigma-Aldrich and used at concentrations of 2 μg/ml and 100 nM, respectively. Wnt3A-conditioned medium was harvested from Wnt3A-expressing L-cells (ATCC, CRL-2647) according to a previously established protocol (Willert et al., 2003). The medium was stored for up to 2 months at 4°C without any detectable loss of Wnt3A activity. Antibodies and molecular probes used for fluorescence microscopy are listed in Table 2.

**Table 2.**
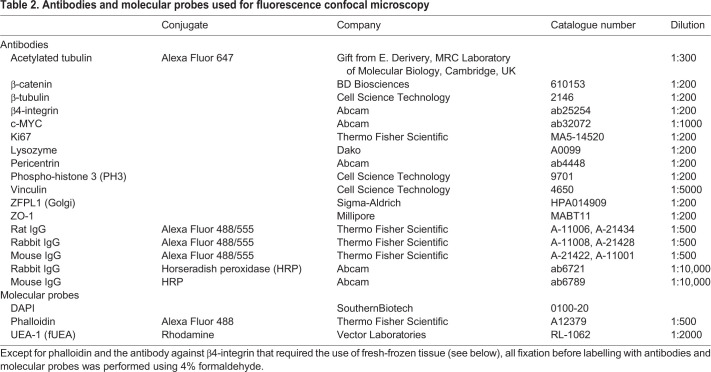
**Antibodies and molecular probes used for fluorescence confocal microscopy**

### Tissue preparation and fluorescent labelling

All procedures using mice were performed under the UK Home Office guidelines. Intestines obtained from wild-type CL57BL/6, *Apc*^1638T/1638T^, *Apc*^Min/+^ CL57BL/6, *Apc*^fl/fl^ *LSL tdTo*m (a gift from the Winton laboratory, CRUK Cambridge Institute, Cambridge, UK) and *Myc-335*^−/−^ (a gift from the Taipale laboratory, Department of Biochemistry, University of Cambridge, UK) mice were either fixed in 4% formaldehyde and embedded in paraffin or fixed-frozen in 10% formalin and embedded in optimal cutting temperature liquid, followed by snap freezing (fresh-frozen tissue).

Small intestinal epithelial sections (4% formaldehyde fixed or fresh frozen) for molecular probe and antibody labelling were cut at 4.5 μm onto slides. The exception was slides labelled with β-tubulin or acetylated tubulin, in which case 20 μm formaldehyde-fixed sections were cut onto poly-L-lysine-coated slides.

For formaldehyde-fixed samples, epitope retrieval was performed in sodium citrate buffer (sodium citrate 10 mM and 0.05% Tween 20, pH 6.0). Primary antibody incubations were carried out at 4°C overnight and secondary antibody incubation for 2 h at room temperature, both in PBS containing normal goat serum (5%) and 0.1% Tween 20. Samples were mounted in DAPI-containing Fluoromount-G (Thermo-Fisher).

### Organoid preparation and fluorescent labelling

Murine small intestinal epithelial organoids were derived from the ileum of mouse small intestine according to Sato and Clevers (2013). Tumouroids were derived from tumours within the ileum of 110 day-old *Apc*^Min/+^ mice (Haigis et al., 2004). All organoids and tumouroids were cultured according to Urbischek et al. (2019).

Organoids were seeded in Matrigel onto eight-well chamber slides (Thermo Fisher Scientific) 48 h before fluorescent labelling, which involved fixation in 92% methanol containing 8% formaldehyde, followed by labelling according to a published protocol (Goldspink et al., 2017). Organoids in primary antibody were incubated at 4°C overnight. The next day the slides were incubated at room temperature for 1 h (allowing the Matrigel to harden), washed and then incubated for 1 h at room temperature in the secondary antibody. Labelled organoid samples were then mounted in DAPI-containing Fluoromount-G.

### Organoid and tissue imaging and data analysis

Fluorescent imaging of tissue was carried out using a Nikon C2 plus confocal microscope using a 40× objective lens. Images were processed using ImageJ software. Fluorescent labelling of each antibody was repeated a minimum of three times.

Imaging of organoids was carried out using a Nikon C2 plus confocal microscope using the 20× and 40× objectives, and an automated spinning disc confocal microscope (Yokogawa Cell Voyager CV8000) using a 40× objective. The *z*-stacks were taken at 1 μm steps. Images were processed and published using ImageJ software. All figures presented are representative images from a single plane within the *z*-stack of the imaged specimen. For the quantification of organelle positioning within organoids, ∼200 cells were manually counted per experiment. Statistical variation of phenotypes among treatment groups and genotypes was calculated by one and two-tailed Student's *t*-tests using GraphPad Prism software. Except where noted, all Student's *t*-tests were unpaired.

### Plasmids and organoid expression

The *piggybac* transposon and Tet-on expression system were kind gifts from Bon-Kyoung Koo (Institute of Molecular Biotechnology, Vienna, Austria). The previously validated shRNA targeting mouse *Apc* (Dow et al., 2015) was inserted into the tet-responsive shRNA expression vector pB-TRE-IRES-mCherry. The three plasmid system also consists of pB-CAG-rtTA, the vector for constitutive rtTA expression, and pPiggybac, the expression vector for constitutive expression of the *piggybac* transposase (Fujii et al., 2015).

The shApc organoid line was generated by transfection of the pB-TRE-shApc-IRES-mCherry, pPiggybac and pB-CAG-rtTA plasmids (Fig. S2A) using a NEPA21 electroporator according to a previously published protocol (Fujii et al., 2015). Organoids were selected for integration of constructs in organoid medium containing Wnt3A-conditioned medium (Urbischek et al., 2019) supplemented with 150 μg/ml Hygromycin B (Thermo Fisher Scientific) for 7 days, after which the medium was switched to organoid medium (Urbischek et al., 2019).

### Validation of shApc organoid line

For western blotting, organoids were recovered from Matrigel using several rinses of ice-cold PBS, and the pellet was lysed with 50 μl 1× RIPA buffer (Millipore) containing protease (Sigma-Aldrich) and phosphatase inhibitors (Roche). Samples were loaded onto NuPAGE 3-8% Tris-acetate gradient gels (Thermo Fisher Scientific) before transfer onto a PVDF membrane. Antibodies used for probing membranes in PBS containing 0.2% Tween 20 and 5% non-fat milk are in Table 2.

Expression levels of Apc and mCherry were determined by qRT-PCR. RNA was isolated from organoids and tumouroids using the ReliaPrep RNA Cell Miniprep System kit (Promega) and cDNA was prepared using the High Capacity cDNA Reverse Transcription kit (Thermo Fisher Scientific), all according to the manufacturers' instructions. qRT-PCR was carried out using Fast SYBR Green Master Mix using a QuantStudio 5 real-time PCR system (both Applied Biosystems). B2m was used as a housekeeping gene and relative fold changes in Apc and mCherry expression were derived from ΔΔCT. The following primers were used: Apc forward, 5′-AGCCATGCCAACAAAGTCATCACG-3′, Apc reverse, 5′-TTCCTTGCCACAGGTGGAGGTAAT-3′; mCherry forward, 5′-CACGAGTTCGAGATCGAGGG-3′, mCherry reverse, 5′-CAAGTAGTCGGGGATGTCGG-3′; and B2m forward 5′-ACCCCCACTGAGACTGATAC-3′, B2m reverse, 5-ATCTTCAGAGCATCATGATG-3.

### Genotyping of *Myc-335^−/−^* mice

Primer pairs used for the genotyping of mice were U3An2 and UcSe2 for wild-type allele, and U3An2 and U5Se2 for the Myc-335 null allele. The sequence of these primers are as follows:

U3An2, 5′-TATCTGCGGGTAGTACACCTGT-3′; U5Se2, 5′-TAGTGATTGGGTAATAAAGAATGAGGTC-3′; and UcSe2, 5′-GCTGACAGAGATTGCTGACATAA-3′ (Sur et al., 2012). The expected amplicon sequences of the two alleles are shown in Table S1.

## Supplementary Material

Supplementary information

Reviewer comments
