# Supplementary material for: Mutational inactivation of Apc in the intestinal epithelia compromises cellular organisation
Source: J Cell Sci. 2021 Jan 27;134(2):jcs250019. doi: 10.1242/jcs.250019 (PMC7860127; doi:10.1242/jcs.250019)
Supplement: Supplementary information [file joces-134-250019-s1.pdf]

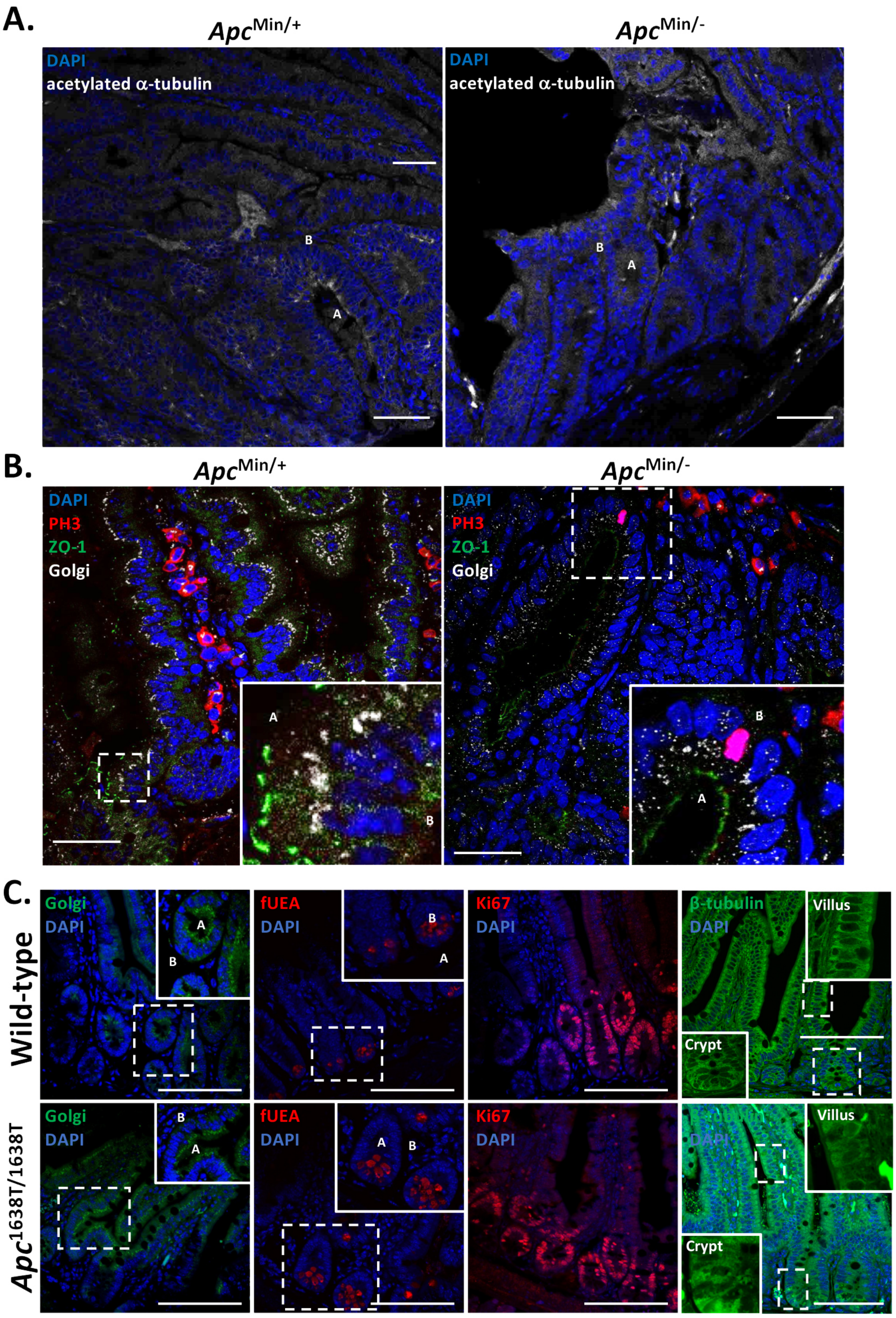


**Figure S1. No loss of microtubule organisation and intestinal epithelial morphology in *Apc*$^{1638T/1638T}$ mice. A.** Fluorescence confocal microscopy of sections of small intestinal epithelia sections from *Apc*$^{Min/+}$ mouse (*left panel*) and *Apc*$^{Min/-}$ tumours (*right panel*) using an antibody to acetylated α-tubulin (grey) and DAPI (blue). "A" marks the apical domain of cells in the monolayers and "B", the basal domain. Scale bars, 50 µm. **B.** *Apc*$^{Min/+}$ epithelial (*left panel*) and *Apc*$^{Min/-}$ tumours (*right panel*) sections were labelled with antibodies to phospho-histone 3 (PH3, red), ZO-1 (green) and ZPLF1 (grey) and DAPI (blue). Scale bars, 50 µm. *C.* Confocal fluorescence images of small intestinal epithelia from a wild-type (*top panels*) and *Apc*$^{1638T/1638T}$ (*bottoms panels*) mouse. Panels, left to right, were labelled in order with – an antibody to ZPLF1, fUEA, an antibody to the Ki67 antigen and an antibody to β-tubulin. All sections were co-labelled with DAPI. "A" marks the apical domain of cells in the monolayers and "B", the basal domain. Scale bars, 100 µm.

Journal of Cell Science • Supplementary information

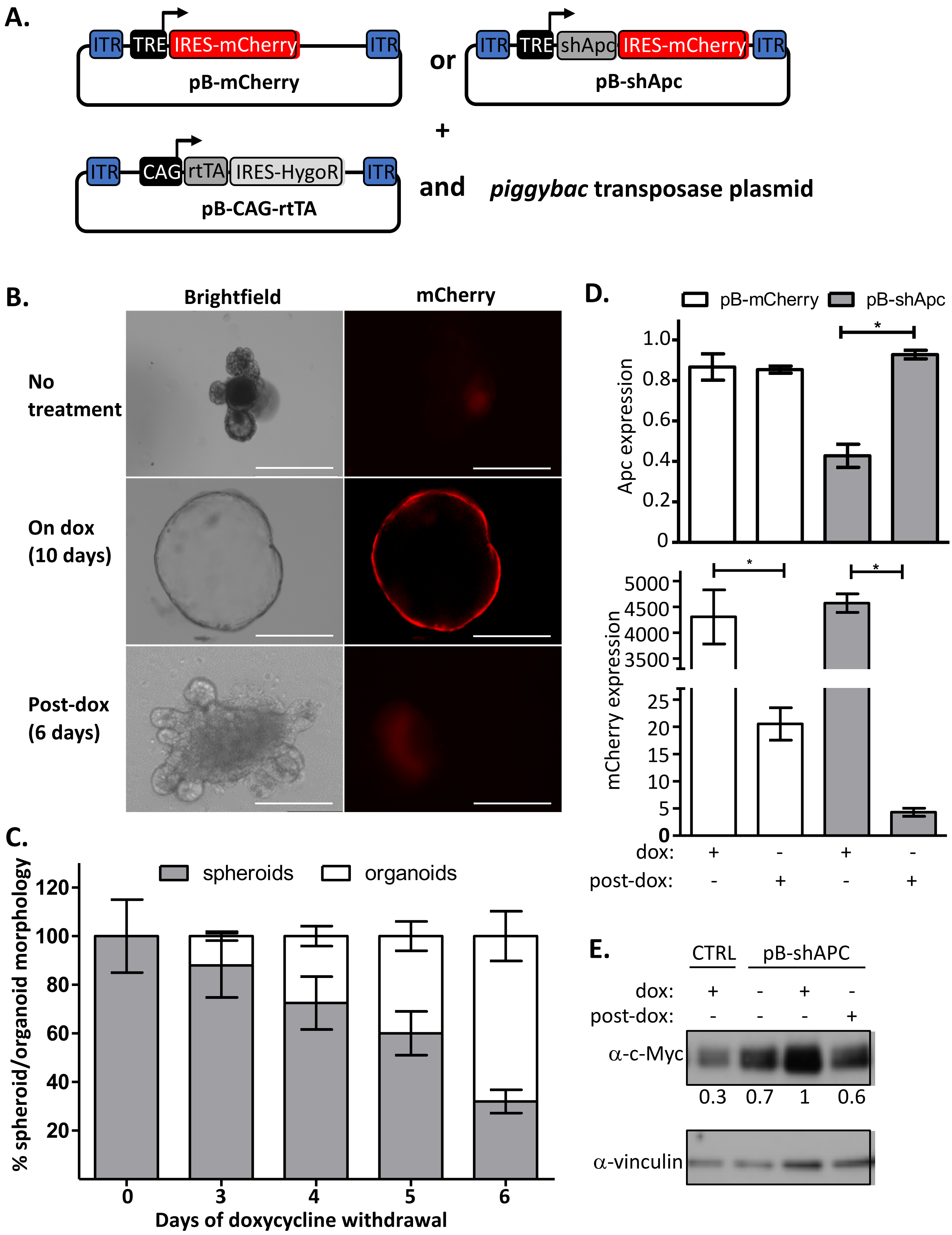


Journal of Cell Science • Supplementary information

**Figure S2. The pB-shApc switchable model of *in vitro* tumorigenesis/tumour regression recapitulates the phenotypic consequences of oncogenic Apc mutations. A.** Transgenes used for the construction of the control pB-mCherry or pB-shApc organoid lines. The shApc expression transgenic system includes pB-CAG-rtTA for constitutive rtTA expression and the plasmid for expression of the *piggybac* transposase for stable integration into organoids. In-built *tet-on* system enables inducible expression of shApc linked to mCherry by treatment of pB-shApc organoids with doxycycline. **B.** Time-course of doxycycline treatment (dox) of pB-shApc organoids; after ten days organoids convert to spheroids accompanied by mCherry expression. Subsequent withdrawal of doxycycline and growth for an additional 6 days (post-dox) restores the budding organoid morphology. Scale bar, 200 µm. **C.** Quantification of the conversion of spheroid morphology to budding organoids morphology upon withdrawal of doxycycline over the course of 6 days. *Grey boxes* represent the remaining spheroids as a percentage of the total; *white boxes* are the number of organoids that have intra-converted from spheroids. Data is derived from greater that 100 organoids from two independent experiments scored at each timepoint. Statistical differences between organoid and spheroid morphologies were evaluated using the two-tailed student's t-test, error bars± SD. **D.** QRT-PCR quantification of relative Apc (*top graph*) and mCherry expression (*bottom graph*) in pB-mCherry control organoids (engineered for inducible expression of mCherry) and pB-shApc organoids (engineered for inducible expression of mCherry and shApc) after two days doxycycline treatment (dox) and at 6-days post-withdrawal (post-dox). Values for Apc and mCherry expression levels are presented with error bars, ± SD. Statistical differences in expression levels after 6-days post-withdrawal relative to doxycycline treated organoids at the $p<0.001$ level of significance is (calculated using a paired Student's *t*-test) represented by '*'. **E.** Protein lysates from control (pB-Cherry) organoids or pB-shApc organoids with various treatments (no treatment, 2 days doxycycline treatment, 6 days post-doxycycline withdrawal; post-dox), shown above, were subject to western blot and probed with antibodies to c-Myc and vinculin as loading control. Quantified relative expression of c-Myc in the lysates were derived from densitometry of the corresponding western blot signals.

Journal of Cell Science • Supplementary information

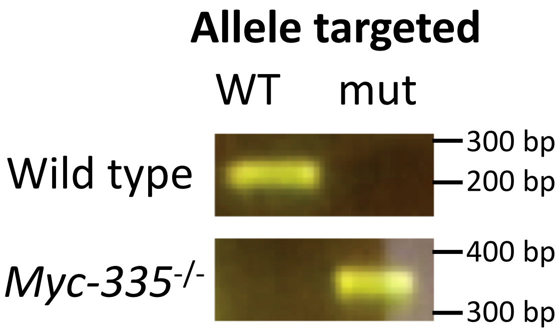


**Figure S3. Genotyping of intestinal epithelia from *Myc-335*$^{-/-}$ mouse used in this study** (Sur et al., 2012). PCR genotyping of *Myc-335*$^{-/-}$intestinal epithelia using primers targeting the wild-type (WT; target amplicon length is 227 bp) or *Myc- 335* mutant allele (mut; amplicon length is 311 bp)(Sur et al., 2012)**.**

**Table S1.** Expected amplicon sequences of the two *Myc-335* alleles.

| **Myc-335$^{wt}$ (227 bp)** | **Myc-335$^{null}$ (311 bp)** |
|---|---|
| GCTGACAGAGATTGCTGACATAAATAAAGTGTG CTCCAAGTGTTTGCTCAGCTCTGGGCTCAGCCAG GACAGATGCAGCTTCTTGAATGTCATTCCCAAGA TATCCTGGAGGTATTTAGCTTCCCTCCCTCTACTC TGGAAAAGATACCATGGGCAACATCAGTACAGA CGATGCTGATATATCCTGACAGAGTATCCCCACT GGACAGGTGTACTACCCGCAGATA | TAGTGATTGGGTAATAAAGAATGAGGTCAAGG AACGGATAATGTAGGGAACAGTGCCTGAGTGT AAGGCAAAAGGCAGAGAATTCCTAGGTTCTAG AACCGGTGACGTCAAGCTCGAATAACTTCGTA TAATGTATGCTATACGAAGTTATTAGGTCCCTC GAAGAGGTTCACTAGTACTGGCCAATCGGCGC GCCTAGGTAGCTTCCCTCCCTCTACTCTGGAAA AGATACCATGGGCAACATCAGTACAGACGATG CTGATATATCCTGACAGAGTATCCCCACTGGAC AGGTGTACTACCCGCAGATA |

Journal of Cell Science • Supplementary information
