# Supplementary material for: Mutational inactivation of Apc in the intestinal epithelia compromises cellular organisation
Source: J Cell Sci. 2021 Jan 27;134(2):jcs250019. doi: 10.1242/jcs.250019 (PMC7860127; doi:10.1242/jcs.250019)
Supplement: Reviewer comments [file joces-134-250019_review_history.pdf]

Mutational inactivation of Apc in the intestinal epithelia compromises cellular organisation

Helena Rannikmae, Samantha Peel, Simon Barry, Takao Senda and Marc de la Roche
DOI: 10.1242/jcs.250019

Editor: Andrew Ewald

Review timeline

Original submission:	10 June 2020
Editorial decision:	28 July 2020
First revision received:	2 November 2020
Editorial decision:	4 December 2020
Second revision received:	7 December 2020
Accepted:	9 December 2020

Original submission

First decision letter

MS ID#: JOCES/2020/250019

MS TITLE: Mutational inactivation of Apc in the intestinal epithelia compromises cellular organisation

AUTHORS: Helena Rannikmae, Samantha Peel, Simon Barry, Inderpreet Kaur Sur, Jussi Taipale, Takao Senda, and Marc de la Roche
ARTICLE TYPE: Research Article

We have now reached a decision on the above manuscript.

To see the reviewers' reports and a copy of this decision letter, please go to: https://submit-jcs.biologists.org and click on the 'Manuscripts with Decisions' queue in the Author Area. (Corresponding author only has access to reviews.)

As you will see, the reviewers are excited by the study and its conclusions but raise a number of substantial criticisms that prevent me from accepting the paper at this stage. They suggest, however, that a revised version might prove acceptable, if you can address their concerns. Cross-referee commenting revealed broad support for the issues raised across all three reviewers. The detailed criticisms are important as are the broad recurring issues of image quality and analysis, APC localization and potential mosaicism, and alternative interpretations of the data. If you think that you can deal satisfactorily with the criticisms on revision, I would be pleased to see a revised manuscript. We would then return it to the reviewers.

We are aware that you may currently be unable to access the lab to undertake experimental revisions. If it would be helpful, we encourage you to contact us to discuss your revision in greater detail. Please send us a point-by-point response indicating where you are able to address concerns raised (either experimentally or by changes to the text) and where you will not be able to do so within the normal timeframe of a revision. We will then provide further guidance. Please also note that we are happy to extend revision timeframes as necessary.

© 2020. Published by The Company of Biologists under the terms of the Creative Commons Attribution License (https://creativecommons.org/licenses/by/4.0/).

Please ensure that you clearly highlight all changes made in the revised manuscript. Please avoid using 'Tracked changes' in Word files as these are lost in PDF conversion.

I should be grateful if you would also provide a point-by-point response detailing how you have dealt with the points raised by the reviewers in the 'Response to Reviewers' box. Please attend to all of the reviewers' comments. If you do not agree with any of their criticisms or suggestions please explain clearly why this is so.

Reviewer 1

Advance summary and potential significance to field

The manuscript by Rannikmae et al examines the function of Adenomatous Polyposis Coli (APC) in intestinal cell polarisation. The authors aim to untangle the potential multifunctional contribution of different APC binding partners and function in intestinal polarisation. This is a long-standing, central question in intestinal tumourigenesis, as APC loss or mutation is thought to be an initiating event in the majority of colorectal tumours. The authors investigate this question using a series of genetically engineered mouse model intestinal sections, or organoids/tumouroids from these models.

The authors are commended for tackling such a fundamental, clinically relevant model using excellent experimental systems. There are, however, some questions about interpretation of the data that undermine the conclusions of the work.

Comments for the author

Main points

1. When using the heterozygous APCMin/+ mice, how do the authors know where this LOH is occurring?
How can these be called APCMin/+ vs APCMin/-. Presumably LOH can happen in a mosaic fashion.

2. Fig. 2A. Why does the orientation of Phalloidin, b4-integrin look inverted? Throughout, the authors compare different orientations of cells, which are not as easily compatible. For instance, transverse sections of villus are compared to longitudinal. Like for like would make interpretation clearer.

3. Fig 2B. The authors are comparing the organisation of b-tubulin in the villi of APCmin/+ mice to the localisation of b-tubulin in the crypt-like structures of the APCMin/- mice. These are not directly comparable. The b-tubulin in the crypt-like structures of the APCMin/- mice look in these images indistinguishable to b-tubulin in the crypts of APCmin/+ mice.

4. Fig 2C. The authors describe a loss of “basal positioning of nuclei, apical positioning of intracellular vesicles and the supra-apical localisation of the Golgi resident protein ZFLP1 and the centrosome marker pericentrin in wild type intestinal epithelia was lost in ApcMin/- tumour cell .”

I fail to see such a loss of polarity in Fig1C. Indeed in the bottom right panel the Golgi in oriented above the nucleus, below a clear apical region marked by ZO-1.

5. Fig 3. A difference is proposed in localisation of the microtubule cytoskeleton between APCMin/+ vs APCMin/- organoids. However, what is compared is the localisation of tubulin in the bud of the APCMin/+organoid to tubulin in the main tumouroid body (from APCMin/-). How are these comparable? In the images presented the tubulin signal in the main organoid body of both genotypes doesn’t appear different.

6. The authors claim that differences in tubulin localisation between APCMin/+ vs APCMin/-, “confirms that compromised epithelial morphology and intracellular disorganisation are a direct consequence of Apc inactivation.”

© 2020. Published by The Company of Biologists under the terms of the Creative Commons Attribution License (https://creativecommons.org/licenses/by/4.0/).

Could another interpretation be that APC is controlling differentiation status not MT organisation directly?
Instead, buds have a different MT organisation to the main organoid body. Upon APC mutation, there is merely a presence vs absence of this particular differentiated morphogenesis. The different MT organisation is representative of that morphogenesis, not differential contribution to MT organisation in the same structures.

7. What are the differences between white and grey bars in Fig S3C?

8. Fig 4B. The staining of the centrosomal marker in the 'Wnt-oid' is unusual. Why is this an almost continual line, rather than puncta?

9. Fig 4C The authors posit that "Apc-dependent control of intracellular organisation and epithelial morphology rely on independent molecular circuits." This is because the authors describe a reversal disruption to Golgi organisation upon nocodazole addition and washout. However, no quantitation is presented for penetrance of phenotype. Similarly, no quantitation is provided to demonstrate that overall 'epithelial morphology' is not altered under these conditions.

Minor points.
- The authors describe that, "We determine that (i) enterocyte proliferation, (ii) microtubule dynamics and (iii) epithelial morphology are controlled by three independent molecular pathways…"

It is not clear from the data exactly how the authors show that these are independent pathways? Could the authors clarify this.

-Line 147-149: "We conclude that, in addition to driving de-regulated epithelial cell proliferation and tissue morphology, Apc inactivation compromises molecular barriers maintaining some aspects of intracellular organisation." What is meant by 'molecular barriers'?

-Line 129. The word 'bulwark' is obscure in English.

-The authors use 'disposition' throughout to describe where something is located. This should be 'position' .

-The authors describe the Golgi as 'supra-apical'. The Golgi is below, not above, the apical membrane, so it should be 'sub-apical'.

-The authors use 'intra-conversion' to describe a shift from one phenotype to another. This should be 'inter-conversion'.

Reviewer 2

Advance summary and potential significance to field

This study explores the role of tumor suppressor Apc in intestinal epithelial morphology and intracellular organization, which is a topic of broad interest to the cell biology community. Using either murine tissue and organoids derived from Apc Min/+ mice, or by silencing Apc in organoids, the authors show evidence of alterations in epithelial morphology, as well as novel findings on altered intracellular organization. They find that microtubules are disorganized, and nuclei, Golgi and centrosome are altered or mislocalised in tumor cells. Authors find that these defects do not depend on C-terminal microtubule and EB1 binding domains of Apc, using a specific mouse model lacking these domains. They further demonstrate that organoids where Wnt pathway is activated by exogenous Wnt addition (Wnt-oids) exhibit cystic phenotype but retain rather normal intracellular organization, unlike Apc Min/- tumoroids, suggesting that Apc affects intracellular organization independently of the Wnt pathway. Also, they show that inhibiting microtubule dynamics disrupts intracellular organization but is not enough to alter established organoid morphology, further indicating that Apc regulation of epithelial morphology is uncoupled from its role in control of intracellular organization (microtubules, centrosome and Golgi). Finally, authors state that Apc

© 2020. Published by The Company of Biologists under the terms of the Creative Commons Attribution License (https://creativecommons.org/licenses/by/4.0/).

regulation of epithelial morphology and intracellular organization by Wnt pathway activity is independent of c-Myc, a key mediator of malignant transformation, whose expression is Wnt pathway-dependent.

Comments for the author

Authors present novel findings on the role of Apc in regulation of intracellular organization by a yet unknown mechanism that is uncoupled from the role of Apc in control of cell proliferation via Wnt pathway and c-Myc.
Even though a clear mechanism is missing, this could potentially merit publication in JCS if a number of concerns, stated below, are addressed; in its present form, I cannot support the manuscript for publication.
My concerns are related to lack of quantitative data, image quality and processing, lack of specific cell markers and data interpretation.

Major concerns:
1. Figures. There are several serious problems with figures.
- Fig. 3A: in the right-most panel (top and bottom), the inset images do not correspond to the boxed regions; in some cases I am not sure whether they are from the same image.
- The same goes for Fig. 3B bottom left and 4A bottom right.
- Fig. 2A: the inset images need to be indicated by boxed regions in the corresponding image, otherwise one cannot see where these insets are coming from. Also, the contrasting/brightness seems to be significantly increased in some inset images, e.g., in Fig. 4A and C. I am not sure if this is an acceptable practice but I believe that the same processing should be applied to the main image and the inset.
2. Image/data quality and interpretation. There are several problems regarding immunostainings (or their lack of) in the figures, which makes it rather challenging to support authors interpretation of the data as in the text.
- It is difficult to assess whether cell alignment is indeed disrupted in Fig. 1 and 2, as markers for cell membrane or junctions (e.g., E-cadherin) are missing. They are necessary in order to visualize cell boundaries and alignment.
- UEA staining is not specific to Paneth cells because it also labels mucus in goblet cells, and this may interfere with the assessment of Paneth cells localization in adenomas and organoids, where crypt-villus morphology is absent or less evident. Thus, I disagree with authors interpretation of these images; to support their interpretation, Paneth cells should be identified with a specific marker (e.g., Lysozyme). Separate figure panels should be made to better identify Paneth cells and assess nuclear shape and cell alignment.
- Additional markers are needed to better infer the mucosal architecture, for example in Fig. 2. Especially in adenomas, it is very hard to infer what is the apical and what is the basal cell pole in these images, and that is indeed very important for this study. In Fig. 3C, it seems that the tight junction marker ZO-1 is also present at the basal side in the control organoids, which is probably due to unspecific staining. This may complicate assessment of polarity in tumoroids, so I suggest to improve the staining procedure or consider another marker/antibody.
- B-tubulin staining in the control tissue (Fig. 2B) does not look very specific to microtubules in this image (it also looks quite different than the same staining in the control organoids (Fig. 3B) which looks more convincing).
Because data on microtubule organization are critical for this paper, this issue should definitely be addressed.
Moreover, in these figures a cell membrane or junctional marker is needed to help discern the boundaries of individual cells and their polarity. The same goes for Fig. 2C, where it is quite difficult to appreciate the described centrosomal defects.
- Fig. 4A: centrosome and Golgi stainings have a significant amount of background, so it is difficult to ascertain the specificity of these stainings. A specific marker for Paneth cells should be used here as well (and not UEA).
3. The paper is overall too descriptive and lacks quantitative data. I think authors should make an effort to provide more quantifications to support their claims, apart from the representative images, especially considering my remarks above. For example, quantification of cell alignment, Paneth cell distribution microtubules organization, nuclei, Golgi and centrosome positioning in the tissue are missing (Figs. 1-2).

© 2020. Published by The Company of Biologists under the terms of the Creative Commons Attribution License (https://creativecommons.org/licenses/by/4.0/).

Together with more convincing images, this should reinforce their conclusions. Also, statistical tests should be employed (lacking in Fig. 3 and 4). Statistical tests used in Fig. 5 are not indicated in the figure legend and in the Methods, thus I am unable to say whether statistics have been used appropriately.
4. Interpretation/conclusions. Line 273 (related to fig. 5): Wnt pathway effect on intracellular organization and tissue morphology is independent of c-Myc expression. Authors used Myc-335-/- mice (Sur et al. 2012), that lack a part of a Myc enhancer, resulting in modestly reduced expression of Myc, according to Sur et al. and resistance to intestinal tumors. Sur and co-authors do not interpret resistance to intestinal tumorigenesis in these mice through a (modest) decrease in Myc expression, and show that only the binding of TCF7l2 transcription factor is abrogated. Thus, it is possible that Myc per se is not responsible for the described absence of tumors in Myc-335-/- mice. If Rannikmae et al. wish to validate or explore the role of potentially altered Myc expression, they should determine Myc protein levels in these mice/organoids.

Minor concerns.
1. Line 123: in addition to deregulated cell proliferation we find..., and also line 316: we did not observe any changes in the rate of proliferation... Authors should present data on cell proliferation, or remove such statements.
2. Related to better images and analysis of microtubular organization, authors should show staining for microtubules in Apc1638T mutant in Fig. S2.
3. line 174: it should probably read supra-nuclear instead of supra-apical.
4. As it is, the abstract sounds too general and some main findings should be mentioned.
5. Line 41: (the key result is to establish..) should be corrected.

<u>Reviewer 3</u>

Advance summary and potential significance to field

The manuscript by Rannikmae et al. increases our understanding of how the tumor suppressor gene adenomatous polyposis coli (Apc) maintains epithelial homeostasis in the murine intestinal epithelia. In this work, the authors made use of adenomas, organoids, and tumoroids derived from the murine model ApcMin/+ to analyze the phenotypic consequences of Apc inactivation. An innovative switchable organoid model of tumorigenesis was generated to determine the immediate effects of silencing Apc. Through experimentations of these model systems, the authors showed that cell proliferation was deregulated, and tissue morphology was disrupted. Other studies have already shown that inactivation of Apc leads to deregulated enterocyte proliferation and compromised tissue organization. However, the authors now have shed some light on how these observations are the direct consequences of oncogenic Apc mutations using complementary and switchable model systems.

The main novelty of this study is that Apc inactivation compromises the microtubule cytoskeleton, leading to the nucleus, Golgi, vesicles, and centrosome disorganization. Although the final model describes facts already known about Apc controlling proliferation and morphology through the Wnt pathway, it was not clear until now if these processes were c-Myc dependent or independent. Besides, the authors have discovered that Apc regulates microtubule cytoskeleton and intracellular organization through Wnt and c-Myc independent pathways. However, these alternative pathways remain unknown. Thus, complementary molecular approaches to unmask this pathway / s and the specific Apc protein regions involved should have increased the interest and scope of the article. Moreover, it would be interesting for the field to analyze in more detail the critical reasons for chromosome instability (CIN) (orientation or positioning of the mitotic spindle, aberrant spindle structures, weaker kinetochore-microtubule attachment, defective spindle checkpoint) using these model systems, accurately the switchable model.

Comments for the author
Specific questions:

-Figure 1. (1B) (ApcMin/-): Although the authors observe that the tumors contain nuclei that do not align along the monolayer's plane, they should have included an extra panel showing it. Moreover, the fUEA signal in ApcMin/- should be more intense.

© 2020. Published by The Company of Biologists under the terms of the Creative Commons Attribution License (https://creativecommons.org/licenses/by/4.0/).

(1C). Domain structure of Apc: Supplemental Figure 1 could be included as part of the main Figure 1C. It is essential to know the different Apc truncations and which one presents with intestinal epithelial tumors.
(1) Indicate if the scheme corresponds to human, mouse, or anything else
(2) Indicate first/last amino acids to understand the length of the wild type and truncated proteins.
(3) Respect to the table below the first domain scheme: they should align the four types of Apc (Min/+, 1638T/1638T, 1322T/+, delta SAMP/+) to the left. The order could be (1) Min/+, (2) 1322T/+, (3) 1638T/1638T, (4) delta SAMP/+. That would definitively help guide the reader.
(4) Please indicate in the scheme where 1322T localizes.
(5) The description of the four “mouse models” should be on the right.
(6) Moreover, the table should include an extra column on the right indicating what is already known/described in the literature about (i) the development of tumors of these mouse models (yes or no) and (ii) the regulation of the Wnt pathway (able, unable), for example.

-Supplemental Figure 1. Although ApcMin/+ is a widely used mouse model to study multiple intestinal neoplasias, the authors must show that there is no signal of Apc (ApcMin/-) by immunohistochemistry, WB (using an antibody which targets the C terminal region of the protein) or qPCR as a control.
-Figure 2. Although the authors describe in the main text, and it is evident in the manuscript, that the actin cytoskeleton is not perturbed, the ApcMin/- DAPI/Phalloidin panel looks very different from the control condition (ApcMin/+). Could the authors show a better image? Moreover, there is no mention of the β-catenin distribution in the main text (lines 162-165).

-Supplemental Figure 2. (1) Align all texts to the top right. (2) All the stains should be more intense. It is hard to see the nuclei in blue (DAPI), and the Golgi signal is weak.

-Figure 3. (3A): The same as Figure 2. The authors do not describe the panel labeled with antibodies to beta-catenin in the main text. Moreover, one of the magnified images (Organoid, β-catenin panel) is not correctly amplified. If the β-catenin localization does not change in Organoids and Tumouroids, Could the authors show similar images?
(3C) Organoid DAPI/fUEL, β4-integrin: the magnified region is difficult to analyze because it is hidden by letters.
(3D) Label the X-axis (cellular organelles, for example). Could it be possible to include in the bar chart the distribution of the same organelles in Organoids as the control condition? If it was100 %, could the authors show it as a red line like in Fig. 4B? or indicate it in the text legend?. A Statistical test to show significance is also required.

-Supplemental Figure 3. (3C) Include legend in the figure (white square: organoids vs. black square: spheroids). (3C, 3D): statistical test to show significance is required. (3D, 3E) Uniformity of terms in order not to mislead reader: +/- Doxycycline, ON, OFF. (3D,3E) the figure 3D is not properly named in the legend, and it does not match (it should be 3E). The same happened to figure 3E (it should be 3D). (3E) Could the authors include the normalized quantification of the c-myc signal? Could they probe the protein lysates with antibodies to Cherry protein as a control? Because this experiment has been done differently from figure 3D (10 days ON/6 OFF vs. two days ON/6 days OFF). Could they probe the protein lysates with actin and tubulin antibodies? As one of the main discoveries of this paper resides in the fact the microtubule cytoskeleton is perturbed, it would be good to show that these proteins' expression does not change. There is no mention of the β-catenin distribution in the main text (lines 203-205).

-Figure 4. Cytoskeleton distribution is a critical issue in the article. Could the authors stain for beta-tubulin and acetylated tubulin? At least for the switchable model and the Wnt-oid/ Tumoriod+Wnt3A. (3B) A Statistical test to show significance is also required.

-Figure 5 is not named correctly in the main text (It is named as figure 5D).

Line 572: Supplementary Figure 4 instead of supplementary Figure 3. Although primers and genotyping conditions are described in (Sur et al., 2012), Could the authors specify the amplicons' length? Based on the conditions described in Sur et al. Wt: 227 bp vs. Myc 335-/-: 352 bp

© 2020. Published by The Company of Biologists under the terms of the Creative Commons Attribution License (https://creativecommons.org/licenses/by/4.0/).

Minor comments:
-It would also be very informative to address the localization of Apc. In lines 74-75, the authors describe that Apc is a substantial protein with many domains but does not detail much about its subcellular location, beyond stabilizing microtubules, controlling cell mobility, and cell division through control of microtubule dynamics. In the end, the authors briefly describe that Apc could contribute to CIN. The absence of this protein causes deregulation of the Wnt pathway and affects CIN, contributing to tumorigenesis. The authors should mention in the text the location of Apc in centrosomes at interface and kinetochores in mitosis, and that it interacts with proteins of the spindle checkpoint.

First revision

Author response to reviewers' comments

We are encouraged that the reviewers recognize that our work addresses a fundamental, clinically relevant problem and that they have offered a very thorough critique of the manuscript. We have addressed all issues raised by the reviewers and this has yielded a more thorough examination of the modes by which Apc regulates intestinal epithelial structure and function. Amended text in the reviewed manuscript is highlighted in red. We address the reviewers' comments, point by point as follows:

Reviewer 1.

1. When using the heterozygous APCMin/+ mice, how do the authors know where this LOH is occurring? How can these be called APCMin/+ vs APCMin/-. Presumably LOH can happen in a mosaic fashion.

In the text, we refer to the mouse model as ApcMin/+; normal tissue is referred to as ApcMin/+ and tumour issue, ApcMIN/- tumours. We have gone through and replaced any instances where this may have caused confusion – for instance line 144 in the original submission.

In the ApcMIN/+ mouse model, LOH in the intestinal epithelia leads to tumours - a mass of Apc deficient cells proliferating independently of niche restrictive cues such as Wnt pathway activators. Previous studies using the ApcMIN/+ mouse model and other models of Apc loss have established that tumours form when both Apc alleles are inactivated in the stem cell compartment (for instance (Barker et al., 2009; Sansom et al., 2004).

It is unlikely that LOH happens in a mosaic fashion, constituent Apc deficient tumour cells will out-compete normal (Apc proficient) cells in tumours where they lack requisite molecular cues instructing them to divide. If the reviewer means whether a tumour can be composed of Apc-deficient cells that have arisen from two or more independent genetic events, we concur – the odds of this happening are low, but it is a possibility. The presence of two or more ApcMIN/- clones in a single tumour will have no bearing on our results or the conclusions of the study.

2. Fig. 2A. Why does the orientation of Phalloidin, b4-integrin look inverted? Throughout, the authors compare different orientations of cells, which are not as easily compatible. For instance, transverse sections of villus are compared to longitudinal. Like for like would make interpretation clearer.

The monolayer of the glandular structures in Apc deficient tumours is uncoordinated and when scoring topology in longitudinal sections we always ensure that our figures capture the correct plane and orientation and we are able to follow the glandular monolayer until it 'closes'. To make the figures clearer, we have labelled apical (A) and basal (B) orientation of the monolayers where appropriate. All sections of wild type tissue are longitudinal, tumour sections do not have defined planes and so our comparison is made to sections where we can evaluate the monolayer of a glandular structure.

© 2020. Published by The Company of Biologists under the terms of the Creative Commons Attribution License (https://creativecommons.org/licenses/by/4.0/).

3. Fig 2B. The authors are comparing the organisation of b-tubulin in the villi of APCmin/+ mice to the localisation of b- tubulin in the crypt-like structures of the APCMin/- mice. These are not directly comparable. The b-tubulin in the crypt-like structures of the APCMin/- mice look in these images indistinguishable to b-tubulin in the crypts of APCmin/+ mice.

We have replaced Fig. 2B with enlarged panels that better represent β-tubulin localisation in ApcMIN/+ epithelia and ApcMIN/- glandular sections. For the ApcMIN/+ section we have included zoomed images of β-tubulin localisation in both villus and crypt monolayers for comparison.

4. Fig 2C. The authors describe a loss of "basal positioning of nuclei, apical positioning of intracellular vesicles and the supra-apical localisation of the Golgi resident protein ZFLP1 and the centrosome marker pericentrin in wild type intestinal epithelia was lost in ApcMin/- tumour cell ."

I fail to see such a loss of polarity in Fig1C. Indeed in the bottom right panel the Golgi in oriented above the nucleus, below a clear apical region marked by ZO-1.

We have replaced the panels for ZFLP1 (Golgi) localisation with new pictures for ApcMIN/+ epithelia to better illustrate the point that the Golgi in ApcMIN/- cells is split apart and mis-localised. One phenotype of Apc loss that we did not develop is that the Golgi splits into smaller punctae – note that the Golgi signal in Fig. 2C is consistently split into smaller punctae that disperse throughout the body of the cells within the tumour monolayer. In certain (rare) instances the Golgi remains supra- nuclear and somewhat clustered in Apc deficient cells consistent with random chance. However, in these rare cases, the Golgi most often fails to recapitulate its normal, centralised disposition.

We have further developed the point by deeper analysis of the microtubule- and actin-cytoskeleton phenotypes in ApcMIN/+ tumours and presented the data in new Table 1.

5. Fig 3. A difference is proposed in localisation of the microtubule cytoskeleton between APCMin/+ vs APCMin/- organoids. However, what is compared is the localisation of tubulin in the bud of the APCMin/+organoid to tubulin in the main tumouroid body (from APCMin/-). How are these comparable? In the images presented the tubulin signal in the main organoid body of both genotypes doesn't appear different.

In Figure 3B we have added a panel showing tubulin staining in both the bud and intervillus space of an organoid – we have ensured that the plane of the confocal section is longitudinally through the middle of the monolayer to accurately demonstrate microtubule localisation and orientation.

6. The authors claim that differences in tubulin localisation between APCMin/+ vs APCMin/-, "confirms that compromised epithelial morphology and intracellular disorganisation are a direct consequence of Apc inactivation."

Could another interpretation be that APC is controlling differentiation status not MT organisation directly? Instead, buds have a different MT organisation to the main organoid body. Upon APC mutation, there is merely a presence vs absence of this particular differentiated morphogenesis. The different MT organisation is representative of that morphogenesis, not differential contribution to MT organisation in the same structures.

We have updated the figures to show that microtubules in tissue and tumour sections and organoids and tumoroids, array along the basal-apical axis of cells and this is apparent in ApcMIN/+ cells from the crypts, villi and intervilli domains of organoids. Compromised microtubule organisation is apparent in ApcMIN/- cells.

7. What are the differences between white and grey bars in Fig S3C?

White boxes are the percentage of budding crypts and grey bars are the percentage of organoids with a spheroid morphology. We have updated the graph and legend for easier interpretation.

© 2020. Published by The Company of Biologists under the terms of the Creative Commons Attribution License (https://creativecommons.org/licenses/by/4.0/).

8. Fig 4B. The staining of the centrosomal marker in the ‘Wnt-oid’ is unusual. Why is this an almost continual line, rather than puncta?

We have replaced the panel with one that better resolves individual centrosomes.

9. Fig 4C The authors posit that “Apc-dependent control of intracellular organisation and epithelial morphology rely on independent molecular circuits.” This is because the authors describe a reversal disruption to Golgi organisation upon nocodazole addition and washout. However, no quantitation is presented for penetrance of phenotype. Similarly, no quantitation is provided to demonstrate that overall ‘epithelial morphology’ is not altered under these conditions.

We have added new panels with bar graphs (Fig. 4D) quantifying the degree of Golgi mis-localisation and effects on organoids morphology (as % crypt maintenance) scored as organoids that maintain greater than 2 or more crypts under the treatment conditions. The figure legend has been updated to reflect this.

Minor points.

- The authors describe that, “We determine that (i) enterocyte proliferation, (ii) microtubule dynamics and (iii) epithelial morphology are controlled by three independent molecular pathways…”

It is not clear from the data exactly how the authors show that these are independent pathways? Could the authors clarify this.

This line was taken from the Abstract. We have added additional text to the Abstract and to the Discussion that elaborates on the three independent effector pathways governed by Apc in the intestinal epithelia to clarify this point.

-Line 147-149:“We conclude that, in addition to driving de-regulated epithelial cell proliferation and tissue morphology, Apc inactivation compromises molecular barriers maintaining some aspects of intracellular organisation.” What is meant by ‘molecular barriers’?

We have changed the two instances where molecular barriers had been used.

-Line 129. The word ‘bulwark’ is obscure in English.

We have changed the instance of bulwark to buttress.

-The authors use ‘disposition’ throughout to describe where something is located. This should be ‘position’ .

We have retained disposition as it refers to the localisation of the organelle in relation to other cell components - position is a less precise term.

-The authors describe the Golgi as ‘supra-apical’. The Golgi is below, not above, the apical membrane, so it should be ‘sub-apical’.

We have changed instances of supra-apical to supra-nuclear or apical and the instance of supra-basally to basal.

-The authors use ‘intra-conversion’ to describe a shift from one phenotype to another. This should be ‘inter-conversion’.

We have retained the term intra-conversion in instances where the individual organoids themselves convert to another form, for instance with our induced Apc depletion studies.

Reviewer 2 Advance Summary and Potential Significance to Field:

This study explores the role of tumor suppressor Apc in intestinal epithelial morphology and intracellular organization, hich is a topic of broad interest to the cell biology community. Using either murine tissue and organoids derived from Apc in/+ mice, or by silencing Apc in organoids,

© 2020. Published by The Company of Biologists under the terms of the Creative Commons Attribution License (https://creativecommons.org/licenses/by/4.0/).

the authors show evidence of alterations in epithelial morphology, as well as novel findings on altered intracellular organization. They find that microtubules are disorganized, and nuclei, Golgi and centrosome are altered or mislocalised in tumor cells. Authors find that these defects do not depend on C-terminal microtubule and EB1 binding domains of Apc, using a specific mouse model lacking these domains. They further demonstrate that organoids where Wnt pathway is activated by exogenous Wnt addition (Wnt-oids) exhibit cystic phenotype but retain rather normal intracellular organization, unlike Apc Min/- tumoroids, suggesting that Apc affects intracellular organization independently of the Wnt pathway. Also, they show that inhibiting microtubule dynamics disrupts intracellular organization but is not enough to alter established organoid morphology, further indicating that Apc regulation of epithelial morphology is uncoupled from its role in control of intracellular organization (microtubules, centrosome and Golgi). Finally, authors state that Apc regulation of epithelial morphology and intracellular organization by Wnt pathway activity is independent of c-Myc, a key mediator of malignant transformation, whose expression is Wnt pathway-dependent.

Reviewer 2 Comments for the Author:
Authors present novel findings on the role of Apc in regulation of intracellular organization by a yet unknown mechanism that is uncoupled from the role of Apc in control of cell proliferation via Wnt pathway and c-Myc. Even though a clear mechanism is missing, this could potentially merit publication in JCS if a number of concerns, stated below, are addressed; in its present form, I cannot support the manuscript for publication. My concerns are related to lack of quantitative data, image quality and processing, lack of specific cell markers and data interpretation.

We have made considerable improvements to the figure datasets and included quantification of phenotypes with Apc deficiency both in figures and in new Table 1.

Major concerns:
1. Figures. There are several serious problems with figures.
- Fig. 3A: in the right-most panel (top and bottom), the inset images do not correspond to the boxed regions; in some cases I am not sure whether they are from the same image.
- The same goes for Fig. 3B bottom left and 4A bottom right.

We have corrected all zoomed images and the corresponding boxes in figures.

- Fig. 2A: the inset images need to be indicated by boxed regions in the corresponding image, otherwise one cannot see where these insets are coming from. Also, the contrasting/brightness seems to be significantly increased in some inset images, e.g., in Fig. 4A and C. I am not sure if this is an acceptable practice but I believe that the same processing should be applied to the main image and the inset.

We have amended the figures accordingly.

2. Image/data quality and interpretation. There are several problems regarding immunostainings (or their lack of) in the figures, which makes it rather challenging to support authors interpretation of the data as in the text.
- It is difficult to assess whether cell alignment is indeed disrupted in Fig. 1 and 2, as markers for cell membrane or junctions (e.g., E-cadherin) are missing. They are necessary in order to visualize cell boundaries and alignment.

We have included new panels in Figure 1B showing β-catenin localisation to delineate cell boundaries and alignment and the position of the nucleus relative to the cells comprising the epithelial and gland structure monolayers. We visualise the nucleus in all figures which serves to guide our assessing of the localisation and disposition of cellular components.

- UEA staining is not specific to Paneth cells because it also labels mucus in goblet cells, and this may interfere with the assessment of Paneth cells localization in adenomas and organoids, where crypt-villus morphology is absent or less evident. Thus, I disagree with authors interpretation of these images; to support their interpretation, Paneth cells should be identified with a specific marker (e.g., Lysozyme). Separate figure panels should be made to better identify Paneth cells and assess nuclear shape and cell alignment.

© 2020. Published by The Company of Biologists under the terms of the Creative Commons Attribution License (https://creativecommons.org/licenses/by/4.0/).

We have detected lysozyme in the intestinal epithelia and tumours and added the figures to our dataset as part of Fig. 1B. We have also amended the text to indicate that fUEA detects vesicles in secretory cells (Paneth and goblet cells).

- Additional markers are needed to better infer the mucosal architecture, for example in Fig. 2. Especially in adenomas, it is very hard to infer what is the apical and what is the basal cell pole in these images, and that is indeed very important for this study. In Fig. 3C, it seems that the tight junction marker ZO-1 is also present at the basal side in the control organoids, which is probably due to unspecific staining. This may complicate assessment of polarity in tumoroids, so I suggest to improve the staining procedure or consider another marker/antibody.

As above, we have visualised ¤-catenin in Fig. 1B to delineate cell boundaries and nuclear position relative to the epithelial and gland structure monolayers allowing us to unequivocally score cell orientation (apical and basal). We have delineated the apical and basal regions in figures, where appropriate.

The ZO-1 detection appearing as basal is in fact apical – it appears as basal because the top of the spherical tumouroid has collapsed during the fixation and staining process and is captured in our confocal images. We have added a note in the results section to this effect.

- B-tubulin staining in the control tissue (Fig. 2B) does not look very specific to microtubules in this image (it also looks quite different than the same staining in the control organoids (Fig. 3B) which looks more convincing). Because data on microtubule organization are critical for this paper, this issue should definitely be addressed.

We have changed the pictures in Fig 2B with higher resolution panels showing the orientation of microtubules along the apical/basal axis and how this localisation is compromised in tumours. We have replaced panels in Fig. 3B to better illustrate this in organoids; the pattern of microtubule arrays along the apical-basal axis of normal epithelial cells is found in both organoids and tissue sections. The pattern is compromised in tumours and tumouroids. Moreover, the signal for acetylated tubulin, normally apical but diffuse upon Apc loss, is consistent in the organoid/tumoroid and epithelia/tumour figures (Fig 3B, Fig S1A).

While we are able to visualise microtubules in epithelial sections and organoids there no effective way to quantify the functional dynamic microtubule cytoskeleton. As we note in the manuscript, we use the localisation of nuclei, intracellular vesicles, the Golgi and centrosome as functional and quantitative markers for the intact microtubule cytoskeleton. New Table 1 summarises our findings.

Moreover, in these figures a cell membrane or junctional marker is needed to help discern the boundaries of individual cells and their polarity. The same goes for Fig. 2C, where it is quite difficult to appreciate the described centrosomal defects.

As mentioned above, we have included a picture of β-catenin localisation (Fig. 1B) relative to nuclear position in the epithelial and glandular monolayers that allows us to score localisation of intracellular components.
In all cases, we have updated figure panels and zoomed images accordingly; for instance, to illustrate the position of the centrosome in Fig. 2C.

- Fig. 4A: centrosome and Golgi stainings have a significant amount of background, so it is difficult to ascertain the specificity of these stainings. A specific marker for Paneth cells should be used here as well (and not UEA).

We have incorporated new panels into figures to better illustrate Golgi positioning in Fig. 4A. We have retained UEA staining in the panel as it adequately demonstrates the phenotype that the alignment of intracellular vesicles within organoids epithelial cells is compromised with Apc loss, supporting our data with centrosome and Golgi localisation. We have modified the text in the manuscript, exchanging the term Paneth cell vesicles for UEA- positive intracellular vesicles.

© 2020. Published by The Company of Biologists under the terms of the Creative Commons Attribution License (https://creativecommons.org/licenses/by/4.0/).

3. The paper is overall too descriptive and lacks quantitative data. I think authors should make an effort to provide more quantifications to support their claims, apart from the representative images, especially considering my remarks above. For example, quantification of cell alignment, Paneth cell distribution, microtubules organization, nuclei, Golgi and centrosome positioning in the tissue are missing (Figs. 1-2).

As per the reviewer's suggestion, we have carried out extensive quantification of the phenotypes we observe throughout the manuscript. We have added new Table 1 contain quantified phenotypic parameters of Apc loss. We have likewise added graphs that quantify parameters in organoids and tumoroids in all figures, where appropriate.

Together with more convincing images, this should reinforce their conclusions. Also, statistical tests should be employed (lacking in Fig. 3 and 4). Statistical tests used in Fig. 5 are not indicated in the figure legend and in the Methods, thus I am unable to say whether statistics have been used appropriately.

We have detailed the statistical test we used (one and two-tailed student t-test) in Materials and Methods and in figure legends as appropriate.

4. Interpretation/conclusions. Line 273 (related to fig. 5): Wnt pathway effect on intracellular organization and tissue morphology is independent of c-Myc expression. Authors used Myc-335-/- mice (Sur et al. 2012), that lack a part of a Myc enhancer, resulting in modestly reduced expression of Myc, according to Sur et al. and resistance to intestinal tumors. Sur and co-authors do not interpret resistance to intestinal tumorigenesis in these mice through a (modest) decrease in Myc expression, and show that only the binding of TCF7l2 transcription factor is abrogated. Thus, it is possible that Myc per se is not responsible for the described absence of tumors in Myc-335-/- mice. If Rannikmae et al. wish to validate or explore the role of potentially altered Myc expression, they should determine Myc protein levels in these mice/organoids.

The reviewer is correct. In consultation with the authors of the 2012 study, Sur and Taipale, we have adjusted our interpretation of the Myc-335 experiment in the Fig. 5 and the results and discussion sections.

Minor concerns.

1. Line 123: in addition to deregulated cell proliferation we find..., and also line 316: we did not observe any changes in the rate of proliferation... Authors should present data on cell proliferation, or remove such statements.

We have measured cell proliferation in organoids/tumoroids by diameter of the organoid/tumouroids shown in Fig. 5D and have added appropriate reference to the manuscript text and Fig. 5 figure legend.

2. Related to better images and analysis of microtubular organization, authors should show staining for microtubules in Apc1638T mutant in Fig. S2.

We have carried out the comparison with wild type tissue and added the figure panels to Fig. S1C

3. line 174: it should probably read supra-nuclear instead of supra-apical.

We have amended throughout the text.

4. As it is, the abstract sounds too general and some main findings should be mentioned.

We have rewritten the abstract to include more detail regarding our main findings.

5. Line 41: (the key result is to establish.) should be corrected.

We have amended the Abstract accordingly.

© 2020. Published by The Company of Biologists under the terms of the Creative Commons Attribution License (https://creativecommons.org/licenses/by/4.0/).

Reviewer 3 Advance Summary and Potential Significance to Field:
The manuscript by Rannikmae et al. increases our understanding of how the tumor suppressor gene adenomatous polyposis coli (Apc) maintains epithelial homeostasis in the murine intestinal epithelia. In this work, the authors made use of adenomas, organoids, and tumoroids derived from the murine model ApcMin/+ to analyze the phenotypic consequences of Apc inactivation. An innovative switchable organoid model of tumorigenesis was generated to determine the immediate effects of silencing Apc. Through experimentations of these model systems, the authors showed that cell proliferation was deregulated, and tissue morphology was disrupted. Other studies have already shown that inactivation of Apc leads to deregulated enterocyte proliferation and compromised tissue organization. However, the authors now have shed some light on how these observations are the direct consequences of oncogenic Apc mutations using complementary and switchable model systems.

The main novelty of this study is that Apc inactivation compromises the microtubule cytoskeleton, leading to the nucleus, Golgi, vesicles, and centrosome disorganization. Although the final model describes facts already known about Apc controlling proliferation and morphology through the Wnt pathway, it was not clear until now if these processes were c Myc dependent or independent. Besides, the authors have discovered that Apc regulates microtubule cytoskeleton and intracellular organization through Wnt and c-Myc independent pathways. However, these alternative pathways remain unknown. Thus, complementary molecular approaches to unmask this pathway / s and the specific Apc protein regions involved should have increased the interest and scope of the article. Moreover, it would be interesting for the field to analyze in more detail the critical reasons for chromosome instability (CIN) (orientation or positioning of the mitotic spindle, aberrant spindle structures, weaker kinetochore-microtubule attachment, defective spindle checkpoint) using these model systems, accurately the switchable model.

Reviewer 3 Comments for the Author: Specific questions:

-Figure 1. (1B) (ApcMin/-): Although the authors observe that the tumors contain nuclei that do not align along the monolayer's plane, they should have included an extra panel showing it. Moreover, the fUEA signal in ApcMin/- should be more intense.

We have amended the figure, raised the intensity of the fUEA signal and added panels to illustrate the consequence of Apc deficiency on the localisation of ¤-catenin and lysozyme. As per the reviewer’s suggestion, we have added alignment of nuclei as part of our phenotypic quantitation panel in Table 1.

(1C). Domain structure of Apc: Supplemental Figure 1 could be included as part of the main Figure 1C. It is essential to know the different Apc truncations and which one presents with intestinal epithelial tumors.
(1) Indicate if the scheme corresponds to human, mouse, or anything else
(2) Indicate first/last amino acids to understand the length of the wild type and truncated proteins.
(3) Respect to the table below the first domain scheme: they should align the four types of Apc (Min/+, 1638T/1638T, 1322T/+, delta SAMP/+) to the left. The order could be (1) Min/+, (2) 1322T/+, (3) 1638T/1638T, (4) delta SAMP/+. That would definitively help guide the reader.
(4) Please indicate in the scheme where 1322T localizes.
(5) The description of the four “mouse models” should be on the right.
(6) Moreover, the table should include an extra column on the right indicating what is already known/described in the literature about (i) the development of tumors of these mouse models (yes or no) and (ii) the regulation of the Wnt pathway (able, unable), for example.

We have taken all suggestions on board, modified the table, updated the figure legend and included it as Fig. 1C.

-Supplemental Figure 1. Although ApcMin/+ is a widely used mouse model to study multiple intestinal neoplasias, the authors must show that there is no signal of Apc (ApcMin/-) by immunohistochemistry, WB (using an antibody which targets the C terminal region of the protein) or qPCR as a control.

As the reviewer notes, the ApcMIN/+ mouse model is well studied and it has been established that

© 2020. Published by The Company of Biologists under the terms of the Creative Commons Attribution License (https://creativecommons.org/licenses/by/4.0/).

the ApcMIN/+ tumours in the model do not express full length Apc, only the truncated form – we have included addition text and references in the introduction to reinforce the point.

-Figure 2. Although the authors describe in the main text, and it is evident in the manuscript, that the actin cytoskeleton is not perturbed, the ApcMin/- DAPI/Phalloidin panel looks very different from the control condition (ApcMin/+). Could the authors show a better image? Moreover, there is no mention of the ¤-catenin distribution in the main text (lines 162-165).

We have modified the figure and included text in the manuscript to better describe the point that, while the morphology of the monolayer is altered with Apc deficiency, orientation and position of the F-actin is maintained.

We have also included new panels in Fig. 1B showing the localisation of b-catenin in normal epithelia and tumour tissue with appropriate reference in the main text.

-Supplemental Figure 2. (1) Align all texts to the top right. (2) All the stains should be more intense. It is hard to see the nuclei in blue (DAPI), and the Golgi signal is weak.

We have updated the figure with the reviewer's suggestions and added new panels to new Fig. S1.

-Figure 3. (3A): The same as Figure 2. The authors do not describe the panel labeled with antibodies to beta-catenin in the main text. Moreover, one of the magnified images (Organoid, β-catenin panel) is not correctly amplified. If the ¤eta- catenin localization does not change in Organoids and Tumouroids, Could the authors show similar images?

The figure has been amended as per reviewers suggestions.

(3C) Organoid DAPI/fUEL, βeta4-integrin: the magnified region is difficult to analyze because it is hidden by letters.

The figure has been amended.

(3D) Label the X-axis (cellular organelles, for example). Could it be possible to include in the bar chart the distribution of the same organelles in Organoids as the control condition? If it was100 %, could the authors show it as a red line like in Fig. 4B? or indicate it in the text legend?. A Statistical test to show significance is also required.

We have amended this panels according to the reviewer's suggestions.

-Supplemental Figure 3. (3C) Include legend in the figure (white square: organoids vs. black square: spheroids). (3C, 3D): statistical test to show significance is required. (3D, 3E) Uniformity of terms in order not to mislead reader: +/- Doxycycline, ON, OFF. (3D,3E) the figure 3D is not properly named in the legend, and it does not match (it should be 3E). The same happened to figure 3E (it should be 3D).

We have amended the figures and legends according to reviewers suggestions.

(3E) Could the authors include the normalized quantification of the c-myc signal?

We have added quantification of the normalised c-Myc signal.

Could they probe the protein lysates with antibodies to Cherry protein as a control? Because this experiment has been done differently from figure 3D (10 days ON/6 OFF vs. two days ON/6 days OFF). Could they probe the protein lysates with actin and tubulin antibodies? As one of the main discoveries of this paper resides in the fact the microtubule cytoskeleton is perturbed, it would be good to show that these proteins' expression does not change.

We have not added western blots for actin and tubulin because of the focus of the study on function of the microtubule cytoskeleton and the actin cytoskeleton. Expression levels of the

© 2020. Published by The Company of Biologists under the terms of the Creative Commons Attribution License (https://creativecommons.org/licenses/by/4.0/).

monomeric tubulin and actin forms fall outside the scope of the study.

There is no mention of the □-catenin distribution in the main text (lines 203-205).

We have added this to the main text.

-Figure 4. Cytoskeleton distribution is a critical issue in the article. Could the authors stain for beta-tubulin and acetylated tubulin? At least for the switchable model and the Wnt-oid/ Tumoriod+Wnt3A.

We have amended the manuscript text to reinforce the fact that there is no reliable metric to quantify the correct pattern of microtubules in organoids. Our study establishes that Apc function controls the microtubule cytoskeleton and in turn intracellular organisation in epithelial cells. Our conclusions on microtubule function rely on the quantification of intracellular vesicles, nuclei, centrosomes and Golgi localisation – see new Table 1.

(3B) A Statistical test to show significance is also required.

We have added text to the legend and Materials and Methods detailing the student's t-test that was used for statistical analysis.

-Figure 5 is not named correctly in the main text (It is named as figure 5D).

We have amended the figure reference.

Line 572: Supplementary Figure 4 instead of supplementary Figure 3. Although primers and genotyping conditions are described in (Sur et al., 2012), Could the authors specify the amplicons' length? Based on the conditions described in Sur et al. Wt: 227 bp vs. Myc 335-/-: 352 bp

We have updated the figure reference. The figure panels and legend for Suppl. Fig. 4 have been amended.

Minor comments:
-It would also be very informative to address the localization of Apc. In lines 74-75, the authors describe that Apc is a substantial protein with many domains but does not detail much about its subcellular location, beyond stabilizing microtubules, controlling cell mobility, and cell division through control of microtubule dynamics. In the end, the authors briefly describe that Apc could contribute to CIN. The absence of this protein causes deregulation of the Wnt pathway and affects CIN, contributing to tumorigenesis. The authors should mention in the text the location of Apc in centrosomes at interface and kinetochores in mitosis, and that it interacts with proteins of the spindle checkpoint.

We have added text to the introduction concerning Apc localisation.

<u>Second decision letter</u>

MS ID#: JOCES/2020/250019

MS TITLE: Mutational inactivation of Apc in the intestinal epithelia compromises cellular organisation

AUTHORS: Helena Rannikmae, Samantha Peel, Simon Barry, Takao Senda, and Marc de la Roche
ARTICLE TYPE: Research Article

We have now reached a decision on the above manuscript.

© 2020. Published by The Company of Biologists under the terms of the Creative Commons Attribution License (https://creativecommons.org/licenses/by/4.0/).

To see the reviewers' reports and a copy of this decision letter, please go to: https://submit-jcs.biologists.org and click on the 'Manuscripts with Decisions' queue in the Author Area. (Corresponding author only has access to reviews.)

As you will see, the reviewers gave favourable reports and are all supportive of publication. There are just a couple of minor issues raised by Reviewer 2 left. I hope that you will be able to carry these out, because I would like to be able to accept your paper.

We are aware that you may be experiencing disruption to the normal running of your lab that makes experimental revisions challenging. If it would be helpful, we encourage you to contact us to discuss your revision in greater detail. Please send us a point-by-point response indicating where you are able to address concerns raised (either experimentally or by changes to the text) and where you will not be able to do so within the normal timeframe of a revision. We will then provide further guidance. Please also note that we are happy to extend revision timeframes as necessary.

Please ensure that you clearly highlight all changes made in the revised manuscript. Please avoid using 'Tracked changes' in Word files as these are lost in PDF conversion.

I should be grateful if you would also provide a point-by-point response detailing how you have dealt with the points raised by the reviewers in the 'Response to Reviewers' box. Please attend to all of the reviewers' comments. If you do not agree with any of their criticisms or suggestions please explain clearly why this is so.

Reviewer 1

Advance summary and potential significance to field

I thank the authors for their thoughtful update to their manuscript, which now addresses all of my previous queries.

Comments for the author

I thank the authors for their thoughtful update to their manuscript, which now addresses all of my previous queries.

Reviewer 2

Advance summary and potential significance to field

The authors have carefully considered my comments and have performed additional work to address my concerns, including immunostaining experiments and image quantifications. The figures are well laid out and previous minor concerns have also been amended.
I am surprised that the authors used beta-catenin as a marker to delineate cell boundaries, considering that it is highly expressed and localised to the nucleus in Apc-deficient cells. However, it appears that it has maintained a sufficient level of junctional localisation to allow the authors to visualise cell boundaries and score localisation of intracellular components.

I find this an interesting and important study, which brings a new understanding of the role of APC in intestinal epithelial architecture and homeostasis, and I am glad to have been able to contribute to peer review of this article.

Comments for the author

Minor comments.
1. I noticed an inconsistency between two sentences on page 7, referring to cell shape in ApcMin/- tumors.

© 2020. Published by The Company of Biologists under the terms of the Creative Commons Attribution License (https://creativecommons.org/licenses/by/4.0/).

"Such ApcMin/- tumours are composed of gland-like structures that maintain a columnar epithelial monolayer..."
"In general, tumour cells fail to maintain the consistent columnar cell shape and size of wild-type epithelial cells..."
It is probably the first sentence that needs to be corrected, as cell shape does not seem to be consistently columnar in these tumors.

2. The ZO-1 staining in Fig. 2C seems to be absent or extremely faint.

Reviewer 3

Advance summary and potential significance to field

The manuscript by Rannikmae et al. increases our understanding of how the tumor suppressor gene adenomatous polyposis coli (Apc) maintains epithelial homeostasis in the murine intestinal epithelia. In the revised manuscript, the authors organized and improved the figures' added panels with new markers, increased intensities and included panel letters to better understand their orientation and organization. They have included statistical analysis and changed some graphics, which now look much more intuitive. They have also modified parts of the text, including some information that was requested. Thus, the authors have shed some light on how these observations are the direct consequences of oncogenic Apc mutations using complementary and switchable model systems. I believe it is now ready for publication in JCellScience.

Comments for the author

The manuscript by Rannikmae et al. increases our understanding of how the tumor suppressor gene adenomatous polyposis coli (Apc) maintains epithelial homeostasis in the murine intestinal epithelia. In the revised manuscript, the authors organized and improved the figures' added panels with new markers, increased intensities and included panel letters to better understand their orientation and organization. They have included statistical analysis and changed some graphics, which now look much more intuitive. They have also modified parts of the text, including some information that was requested. Thus, the authors have shed some light on how these observations are the direct consequences of oncogenic Apc mutations using complementary and switchable model systems. I believe it is now ready for publication in JCellScience.

Second revision

Author response to reviewers' comments

Reviewer 2 Comments for the Author:
Minor comments.

1. I noticed an inconsistency between two sentences on page 7, referring to cell shape in ApcMin/- tumors."Such ApcMin/- tumours are composed of gland-like structures that maintain a columnar epithelial monolayer..."
"In general, tumour cells fail to maintain the consistent columnar cell shape and size of wild-type epithelial cells..."
It is probably the first sentence that needs to be corrected, as cell shape does not seem to be consistently columnar in these tumors.

We have changed the sentence "Such ApcMin/- tumours are composed of gland-like structures that maintain a columnar epithelial monolayer..." to "Such ApcMin/- tumours are composed of gland-like structures that maintain an epithelial monolayer...".

2. The ZO-1 staining in Fig. 2C seems to be absent or extremely faint.

© 2020. Published by The Company of Biologists under the terms of the Creative Commons Attribution License (https://creativecommons.org/licenses/by/4.0/).

We have raised the brightness of the ZO-1 staining in Fig. 2C for better visibility

---

Third decision letter

MS ID#: JOCES/2020/250019

MS TITLE: Mutational inactivation of Apc in the intestinal epithelia compromises cellular organisation

AUTHORS: Helena Rannikmae, Samantha Peel, Simon Barry, Takao Senda, and Marc de la Roche
ARTICLE TYPE: Research Article

Thank you for making those two small changes quickly. I am happy to tell you that your manuscript has been accepted for publication in Journal of Cell Science, pending standard ethics checks. I verified the changes myself and did not send it for review.

© 2020. Published by The Company of Biologists under the terms of the Creative Commons Attribution License (https://creativecommons.org/licenses/by/4.0/).
